# Selective and metal-free epoxidation of terminal alkenes by heterogeneous polydioxirane in mild conditions

**DOI:** 10.1098/rsos.171541

**Published:** 2018-05-02

**Authors:** M. Kazemnejadi, A. Shakeri, M. Nikookar, R. Shademani, M. Mohammadi

**Affiliations:** 1 Department of Chemistry, College of Sciences, Golestan University, Gorgan, Iran; 2 Faculty of Chemistry, University College of Science, University of Tehran, Tehran, Iran; 3 Department of Marine Technology, Amirkabir University of Technology, Tehran, Iran

**Keywords:** epoxidation, polysalicylaldehyde, polydioxirane, terminal alkene, recoverability, mild conditions

## Abstract

Polydioxirane (PDOX) was prepared by the treatment of polysalicylaldehyde with Oxone and was found as a selective, highly efficient and heterogeneous reagent for epoxidation of alkenes which can be successfully isolated. This work also introduced a simpler, safer and milder way for epoxidation of alkenes with dioxirane groups than before. PDOX can be simply recovered from the reaction mixture by plain filtration and reused for eight runs without significant reactivity loss.

## 1. Introduction

Epoxidation of alkenes as a fundamental reaction in organic synthesis provides oxirane (epoxide) compounds that are considered as useful synthetic intermediates in the production of epoxy resins, perfumes, plasticizers, pharmaceuticals, cosmetics, etc. [1–4]; but their production involves high consumption of energy, lack of selectivity and environmental concerns [5,6]. So, finding a highly selective method is in demand for the preparation of such materials.

Chemistry of dioxirane compounds as powerful oxidants, whether isolated or *in situ* generated, has been well known for decades [7,8]. Dioxirane compounds as non-metal organic oxidants can efficiently transfer oxygen atoms to various substrates and their utility has been well known for various oxidation reactions such as epoxidation of various olefins (enol ether, silyl enol ether, 2,3-dimethylbenzofuran, enol ester, lactone and enol phosphate) [9], oxidation of sulfides, amines and carbon–hydrogen bond [10]. Among them dimethyldioxirane (DMDO), known as Murray's reagent [11,12], is an oxidant used in organic synthesis and considered as an environment-friendly oxygen transfer reagent for suitable substrates without the need for a catalytic system containing transition metal [13–15]. However, DMDO is susceptible to decomposition especially in the presence of heat; it should be isolated at lower temperatures mostly as acetone solution. One strategy to overcome these problems is the heterogenization of homogeneous catalysts by grafting or anchoring dioxirane groups on a solid surface, or by immobilization on polymers or porous materials [16]. Previously, Satori *et al.* [17] reported heterogeneous solid catalysts by supporting α-fluorotropinone on SiO_2_ and Merrifield resin for the stereoselective epoxidation of alkenes with Oxone. Silica-supported trifluoromethyl ketone was another approach for dioxirane-mediated epoxidation of alkenes in the presence of Oxone, which provides a reusable heterogeneous catalyst [18,19]. Also, D'Accolti *et al.* [20] addressed the application of homogeneous and heterogeneous dioxiranes isolated and generated *in situ* in a review article.

Salicylaldehyde is one of the safe, readily available organic compounds from a health and reactivity point of view [21], which can be extracted from natural sources such as buckwheat [22]. In this work, we used an efficient method for polymerization of salicylaldehyde by polycondensation reaction of 2-hydroxy-5-chloromethylbenzaldehyde [23,24].

To overcome the drawbacks for DMDO, herein, for the first time we decorated oxirane pendant groups on polysalicylaldehyde (PSA) as a polymer support, which could be readily prepared, isolated as powder and characterized, and can efficiently catalyse the epoxidation of alkenes in a heterogeneous protocol with possibility of recovery. Decorating of dioxirane groups on polymer leads to an increase in stability for different studies.

## 2. Material and method

### 2.1. Materials and instrumentation

All materials were purchased from Sigma Aldrich and Merck and used as received without any purification. Fourier transform infrared (FTIR) spectra were obtained using a Bruker EQUINOX 55 FT-IR spectrophotometer using KBr pellets. Analytical thin layer chromatography (TLC) was carried out on glass plates covered with silica gel. The ^1^H NMR spectra were recorded in DMSO-*d*_6_ and CDCl_3_ using a Bruker Avance AQS 300 MHz. All chemical shifts are reported in δ units downfield from tetramethylsilane. Elemental analyses (C, H, N) were performed on a Perkin Elmer-2004 instrument. UV–visible analyses were performed on UV Spectrolab BEL photonics. Thermal studies (TGA-DTG) have been performed on a NETZSCH STA 409 PC/PG in nitrogen atmosphere with a heating rate of 20°C min^−1^ in the temperature range of 25–750°C. Gel permeation chromatography (GPC) was conducted with a Knauer advanced scientific instrument, Germany, with RI detector (Smartline 2300) PL gel 10 µm, 10E3 A° column. Monodispersed poly(methyl methacrylate) standards were used for calibration. Injected volume was 20 µl. Melting points were measured using Electrothermal IA 9000 melting point apparatus. The products were quantified with a Shimadzu 14B gas chromatograph (GC) equipped with a 3 m Silicon DC-200 packed column and a flame ionization detector, along with 1,2-dichlorobenzene as an internal standard. The concentrations of each organic product were calibrated relative to that of an internal standard.

### 2.2. Preparation of polysalicylaldehyde **2**

**Scheme 1.**
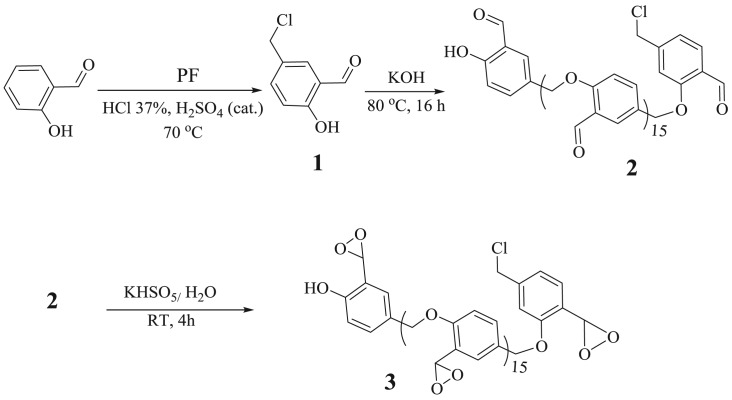
Preparation of PSA **2** and PDOX **3**.

2-Hydroxy-5-chloromethylbenzaldehyde **1** was synthesized and purified according to a previously described procedure [24]; salicylaldehyde (10 mmol), paraformaldehyde (0.49 g, 16.4 mmol) and HCl 37% (80 mmol) along with several drops of concentrated H_2_SO_4_ as a catalyst were mixed together at 70°C and stirred for 20 h (scheme 1). The reaction mixture was cooled to room temperature, then water (20 ml) was added to the mixture and the product was extracted into CH_2_Cl_2_ (20 ml). Some anhydrous Na_2_SO_4_ was used for drying the organic phase. Remaining CH_2_Cl_2_ was removed with a rotary evaporator. A pale purple product **1** as powder was isolated (9.5 mmol, 95% yield). Polymerization of **1** (scheme 1) (6.0 mmol) was done in the presence of concentrated KOH (50% (v/v), 20 mmol) at 80°C which after stirring for 16 h afforded a dark yellow solid (PSA, **2**). The mentioned solid was filtered and washed with distilled water (3 × 10 ml) to complete elimination of acid and inorganic salt impurities and then put in an oven (50°C) overnight. Final mass for PSA **2** was 0.83 g (m.p. > 400°C).

### 2.3. Preparation of polydioxirane **3**

In a 100 ml round bottom flask containing an aqueous solution (water buffered at pH 7–7.5, due to auto-decomposition of Oxone at high pH) [25] of sodium bicarbonate (0.85 g, 10.0 mmol in 10.0 ml of deionized water), PSA (0.5 g) was added and the mixture stirred at room temperature. Owing to the production of gas during the preparation, potassium monoperoxy sulfate (Oxone, 6.15 g, 10 mmol) was added in portions (5 × 1.23 g) every 10 min and the reaction stirred for 4 h. Then the reaction mixture was filtered and the solid (polydioxirane, PDOX) collected on paper. PDOX was rinsed with cold acetone (3 × 5 ml) and deionized water (3 × 5 ml) then dried in vacuum. Resultant grey PDOX powder was isolated (0.56 g) and stored as a stable powder in a refrigerator (in accordance with TGA analyses) without any need for specific protection. PDOX gradually was transferred to PSA at room temperature after a week.

### 2.4. Determination of dioxirane groups in polydioxirane

The following procedure (according to the previously described procedure) [26] was used to measure dioxirane groups formed on PDOX. In a 1 ml volumetric test tube, a 0.2 M solution of dimethyl sulfide in deuterated chloroform (CDCl_3_) was treated with 0.5 g PDOX at room temperature and the reaction stirred for 4 h. A portion of the solution was added directly to an NMR tube. The analysis of the ^1^H NMR via signal integration of the dimethyl sulfoxide protons at approximately 2.0 ppm against the dimethyl sulfide protons at approximately 2.5 ppm allows the determination of dioxirane groups in PDOX.

### 2.5. General procedure for stepwise polydioxirane epoxidation of alkenes

In a typical run for stepwise epoxidation of alkenes by PDOX **3**, to a 25 ml round bottom flask, 0.3 g of PDOX (containing 2.25 mmol dioxirane group) was added to an anhydrous CH_2_Cl_2_ (10 ml) solution of alkene (2.0 mmol). The mixture was stirred at room temperature (heterogeneous medium). After completion of the reaction as indicated by TLC (2–8 h), the catalyst was filtered off and the solvent was evaporated to give epoxide product. Flash chromatography was used in some cases for further purification. The recovered reagent (that is now PSA) was washed with distilled water (3 × 5 ml) and acetone (3 × 5 ml) and stored in a refrigerator for the next reaction (scheme 1).

### 2.6. General procedure for *in situ* polydioxirane epoxidation of alkenes

Epoxidation of alkenes was also accomplished by *in situ* preparation of PDOX in the mixture using the following procedure. Alkene (2.0 mmol) was dissolved in 25.0 ml of CH_2_Cl_2_. Then 10 mg of tetra-*n*-butylammonium hydrogen sulfate, as an oxidizing agent, was dissolved in water (20 ml) and added to the reaction mixture. The pH value of the reaction mixture was monitored and adjusted to 7.5 by the addition of phosphate buffer (10 ml). An amount of 0.3 g of PSA followed by a saturated solution of NaHCO_3_ was added to the reaction mixture. Oxone powder as an oxygen source (1.9 g, 3 mmol) was added in portions. The reaction mixture was stirred vigorously while maintaining the pH of the solution at 7.0–7.5 (heterogeneous medium). Upon completion of the reaction monitored by TLC, PSA was filtered and the remaining solution poured into a separating funnel and diluted with one volume of ethyl acetate. The layers were separated and the organic layer was washed with H_2_O, dried over K_2_CO_3_, then evaporated at reduced pressure (aqueous layer was discarded). The crude product was purified by flash chromatography on silica pretreated with triethylamine using dry loading.

## 3. Results

### 3.1. Characterization of polysalicylaldehyde and polydioxirane

The average molecular weight of PSA was measured through acylation of hydroxyl groups (end group analysis); an analytical method that determined the hydroxyl number (%OH) by the following equation [27,28]:

4.1 $$\%\text{OH}=\frac{(V_{1}-V_{2})\times f\times 0.0085\times 100}{m_{p}},$$

where *V*_1_ (ml) and *V*_2_ (ml) are consumed volumes of potassium hydroxide required for titration of blank and polymer sample, respectively, *f* is the coefficient of 0.5 N KOH solution that is equal to 0.732 [29] and *m*_p_ (g) is the mass of polymer sample weighed for titration. The molecular weight obtained by this method will be numerical molecular weight (${\bar{M}}_{n}$) that was measured by insertion of %OH into the following equation [27,28]:

4.2 $${\bar{M}}_{n}=\frac{56.11\times 1000}{\%\text{OH}}.$$

To measure the hydroxyl number of PSA, 0.017 g of PSA was weighed and dissolved in pyridine/acetic anhydride mixture (88 : 12) along with stirring at 50°C for 8 h. One or two droplets of phenolphthalein indicator were added to the reaction mixture then titrated with KOH (0.5 N). Hydroxyl number of PSA was measured as 25.62 (*V*_2_ *– V*_1_ = 0.7 ml, equation (4.1)). So, ${\bar{M}}_{n}$ was calculated by equation (4.2) as equal to 2190 for PSA.

The molecular weight of the PSA also was studied with GPC. The results obtained from GPC were tabulated (table 1). The numerical molecular weight (${\bar{M}}_{n}$) obtained by GPC was equal to 2226. Small differences (just 36 units) between analytical and instrumentational measuring (GPC) of ${\bar{M}}_{n}$ of PSA exhibited the accuracy of the methods as well as validity of the obtained amount for ${\bar{M}}_{n}$. PSA had a low polydispersity index (PDI = 1.43). An explanation of this low PDI was step-growth polymerization in a concentrated alkali medium which neutralized HCl molecules that formed resulting in condensation of the monomers and playing the role of a driving force for the forward progress of the polymerization reaction. The molecular weight gained from PSA clearly confirms the formation of PSA through polycondensation reaction.

**Table 1.** Data obtained from GPC analysis of PSA.

types of molecular weight		
weight average molecular weight	2278	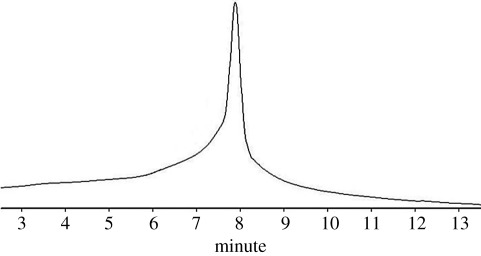
number average molecular weight	2226	
*Z* average molecular weight	2328	
*Z* + 1 average molecular weight	2377	
polydispersity index	1.430	
peak molecular weight	2244	
*Z* average/weight average	1.028	
*Z* + 1 average/weight average	1.056	

**Figure 1.**
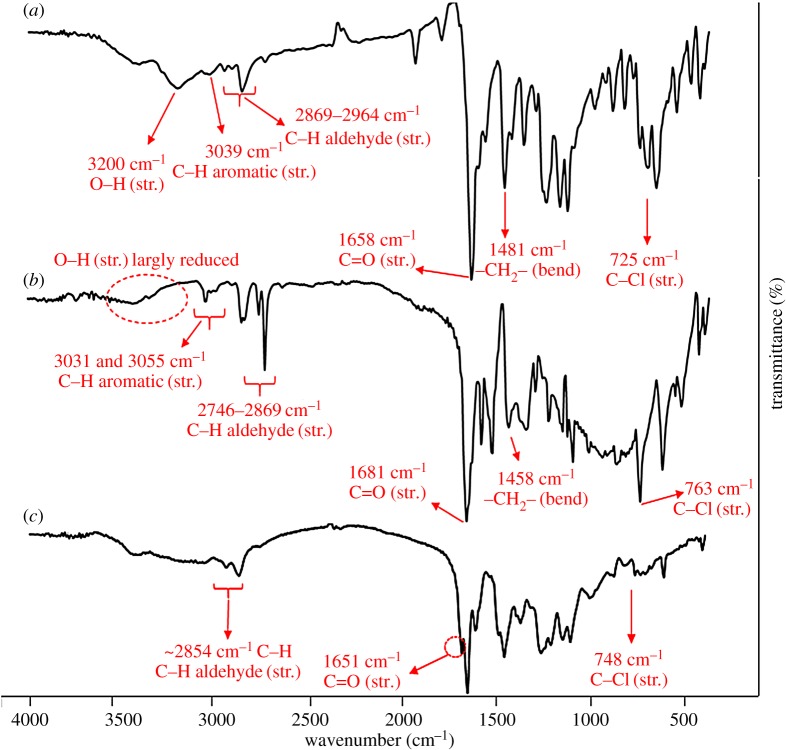
FTIR spectra of 2-hydroxy-5-chloromethylbenzaldehyde **1** (*a*), PSA **2** (*b*) and PDOX **3** (*c*).

FTIR spectra of **1**, **2** and **3** are shown in figure 1. The advent of a peak at 1481 cm^−1^ in the spectrum of **1** was related to the bending vibration of methylene groups (–CH_2_–) in *m*-substitution of aldehyde group (figure 1*a*). Also, the spectra show C–H bending vibrations between 1400 and 1480 cm^−1^ for all the samples. Another characteristic peak for **1** was 725 cm^−1^ for stretching vibration of C–Cl which demonstrated the successful formation of chloromethylene substitution on salicylaldehyde. The presence of C–Cl vibration in the all spectra in the region of approximately 725–763 cm^−1^ demonstrated chloromethylene groups in the polymer terminal chains (scheme 1). Also the existence of two peaks at 800–900 cm^−1^ was assigned to 1,2,4-trisubstituted pattern on the benzene ring. A series of vibrations below 3000 cm^−1^ represented the aldehyde C–H stretching (figure 1) which confirmed the presence of aldehyde groups for all samples. Vibration of C–H for benzene rings was found at 3000–3040 cm^−1^ for the samples. Stretching vibration of hydroxyl groups at 3200 cm^−1^ has been reduced (figure 1*b*, dotted circle) which indicated the formation of ether bonds due to polymerization of salicylaldehyde. The spectra clearly demonstrated elimination of aldehyde groups due to transformation to corresponding dioxirane groups (figure 1*c*, dotted circle).

Physical properties of the samples including their elemental analysis and colours are given in table 2. Elemental analysis also proved the formation of 2-hydroxy-5-chloromethylbenzaldehyde **1** (table 2). The decrease in carbon percentage of PDOX afforded an evidence for the formation of dioxirane groups on PSA.

**Table 2.** Elemental analysis results and colours of the compounds.

		elemental analysis		
compounds	colour	%C	%H	%N
2-hydroxy-5-chloromethylbenzaldehyde	purple	56.82 (56.33)^a^	4.25 (4.14)	—
PSA	yellow	70.11 (70.63)	4.35 (4.48)	—
PDOX	grey	63.34 (63.19)	4.11 (4.01)	—

^a^Theoretically calculated.

**Figure 2.**
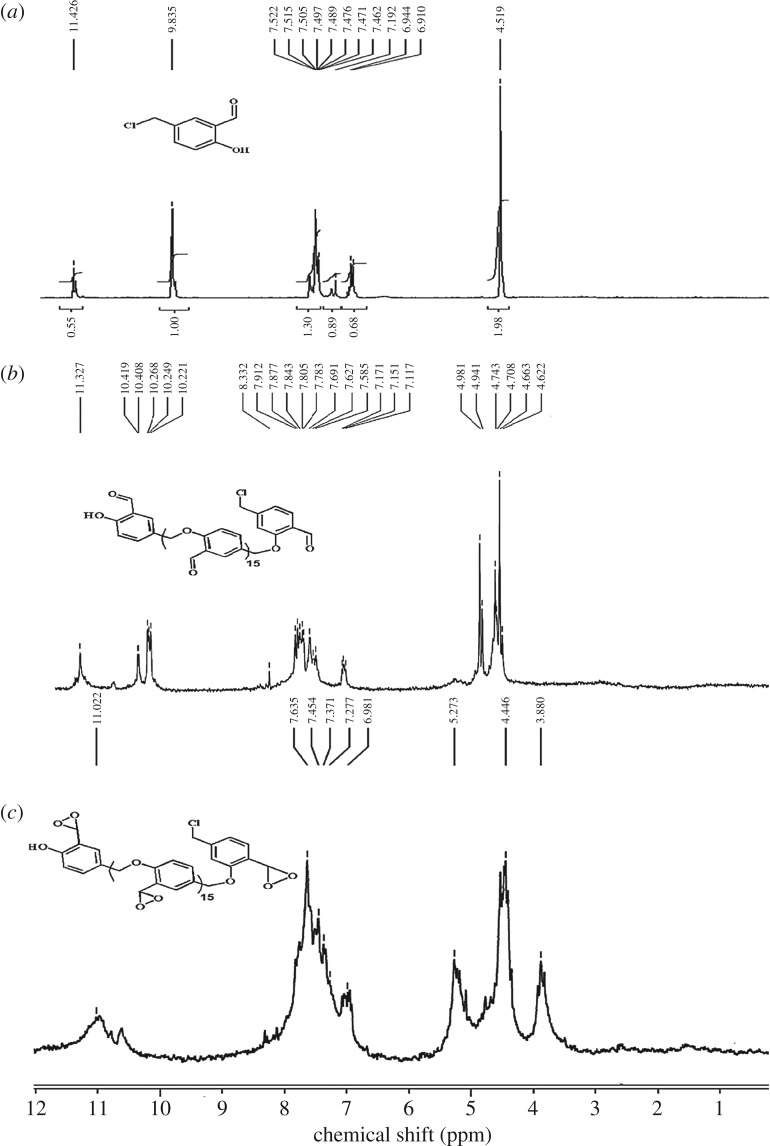
^1^H NMR spectra of (*a*) 2-hydroxy-5-chloromethylbenzaldehyde **1**, (*b*) PSA **2** and (*c*) PDOX **3**.

^1^H NMR spectra of 2-hydroxy-5-chloromethylbenzaldehyde **1**, PSA **2** and PDOX **3** are shown in figure 2. Owing to presence of two directing groups on the phenyl, the chloromethylene group in **1** was substituted at *meta* position as shown in scheme 1 [30]. Resonance for methylene protons for **1** was found at 4.51 ppm. Methylene protons for PSA **2** were represented with three peaks at 4.09, 4.74 and 4.98 ppm. The mentioned peaks appeared in the region of 3.8–5.2 ppm for PDOX. Aromatic protons were set in the region of 6.97–7.57 ppm for **1**, 7.11–8.30 ppm for PSA **2** and 6.98–7.63 ppm for PDOX **3**. Chemical shifts for aldehyde and hydroxyl protons were found at 9.09 and 11.06 ppm for **1**. ^1^H NMR spectrum of PSA shows two peaks for aldehyde at 10.22 and 11.40 ppm due to different chemical environments. Hydroxyl groups at terminal PSA chains exhibit a single peak at 11.33 ppm (figure 2*b*). Hydrogen resonances of dioxirane groups were found at approximately 7.6 ppm as sharp peak which overlapped with resonance of phenyl hydrogens. Furthermore, resonances of the aldehyde hydrogens completely disappeared at 10 ppm due to their transformation to dioxirane groups.

**Figure 3.**
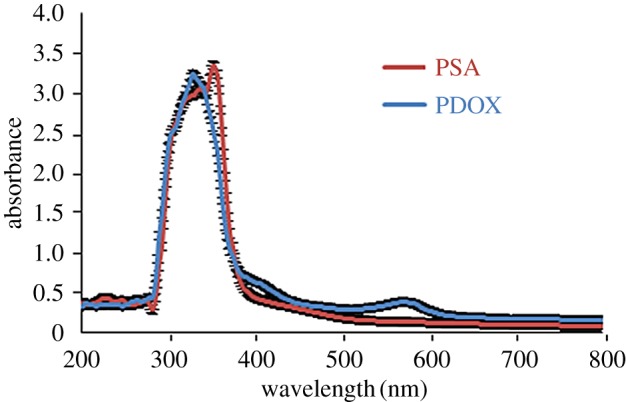
UV–visible spectra of PSA and PDOX.

Figure 3 depicts electronic spectra for PSA and as-synthesized PDOX. PSA showed two adsorptions at 340 nm and 354 nm attributed to π → π* (benzene rings) and *n *→ π* (carbonyl groups). Electronic spectrum of PDOX exhibited a single peak with a maximum of 334 nm; this peak due to transformation of carbonyl compounds to dioxirane groups was assigned to π → π* adsorptions related to benzene rings [9].

**Figure 4.**
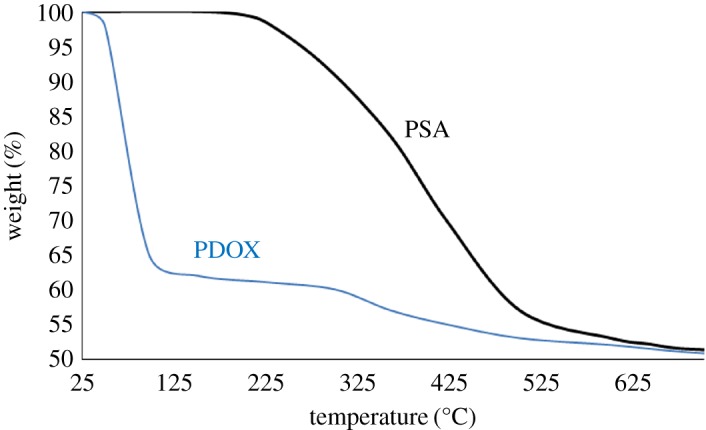
TGA curves of PSA (black) and PDOX (blue).

As indicated in figure 4, TGA weight loss of PSA takes place in one step (temperature span of 200–525°C corresponds to 47% weight loss), while that of PDOX takes place in two steps. A large weight loss (corresponding to 16%) in the beginning (50–62°C) of PDOX TGA curve was attributed to decomposition of dioxirane groups on PSA framework. For this reason, it is recommended that PDOX be stored in a refrigerator. The second step in thermal programme of PDOX that starts at 300°C indicated decomposition of PSA. After that, the weight loss continues to 700°C with a gentle slope, which shows carbonization of the polymer.

Another evidence for the formation of PDOX (or formation of dioxirane groups) was obtained from dimethyl sulfide assay (§3.2) used for concentration determination of dioxirane groups which afforded 7.5 mmol g^−1^.

### 3.2. Determination of dioxirane groups in polydioxirane

**Figure 5.**
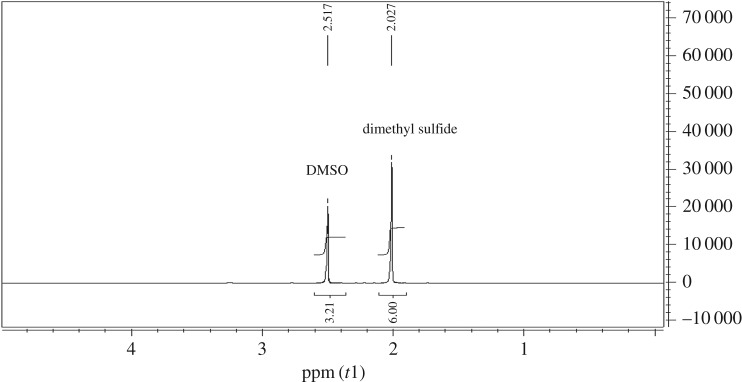
^1^H NMR spectrum results in oxidation of excess dimethyl sulfide in the presence of PDOX.

Commonly, the concentration of dioxirane groups in DMDO can be determined by various methods including iodometric titration, selected ion monitoring, oxidation of methylphenyl sulfide [25,31] or dimethyl sulfide and UV–visible technique [32]. We measured the dioxirane groups by ^1^H NMR technique through reaction of known weight of PDOX with excess dimethyl sulfide. Then concentration of dioxirane groups on PDOX was determined by comparison between signal integration of the DMSO versus signal integration of dimethyl sulfide [32]. The results showed the loading amount of dioxirane groups was 7.5 mmol g^−1^ (figure 5).

## 4. Discussion

Styrene was selected as the model substrate in order to investigate parameters in the reaction. With regard to this, we have investigated the influence of temperature, amount of PDOX (mmol of dioxirane groups) and solvent on epoxidation of styrene.

**Figure 6.**
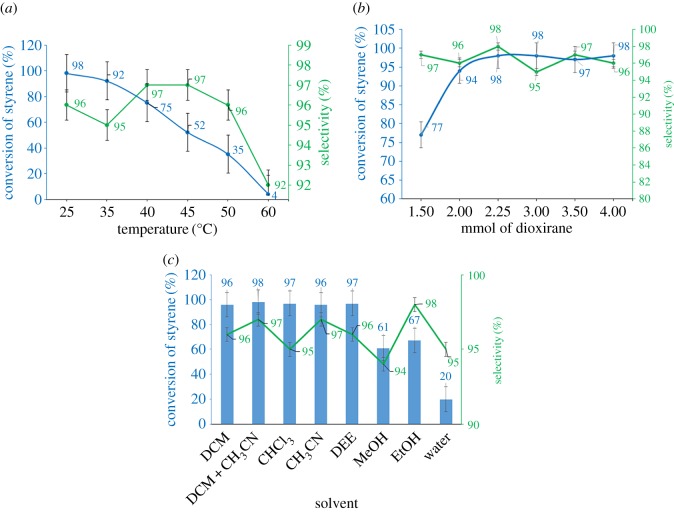
Screening (*a*) temperature, (*b*) PDOX amount and (*c*) solvent type on epoxidation of styrene in the presence of PDOX for 2 h. Green curves demonstrate epoxide selectivity for each study. Reaction conditions: (*a*) stepwise epoxidation: 0.30 g PDOX (containing 2.25 mmol dioxirane group), anhydrous CH_2_Cl_2_ (10 ml) solution of styrene (2.0 mmol), temperature, 2 h. (*b*) Stepwise epoxidation: PDOX, anhydrous CH_2_Cl_2_ (10 ml) solution of styrene (2 mmol), room temperature, 2 h. (*c*) Stepwise epoxidation: 0.30 g PDOX (containing 2.25 mmol dioxirane group), solvent (10 ml), styrene (2 mmol), room temperature, 2 h.

According to TGA results, as expected, due to decomposition of dioxirane groups in temperature span of approximately 50–62°C, raising temperature causes a decrease in epoxide conversion (figure 6*a*). The conversion at 60°C was 4% for 2 h (figure 6*a*). Also, styrene epoxide selectivity decreased slowly with increasing temperatures up to 60°C.

Stoichiometric amount of PDOX (as dioxirane concentration) was found as the optimum amount for gaining the highest possible conversion. Figure 6*b* shows a linear decrease for conversion of styrene with a decrease in stoichiometric amount of PDOX. On the other hand, raising the amount of PDOX did not show any considerable effect on reaction conversion (figure 6*b*).

To elucidate the performance of PDOX for epoxidation reaction, we checked the reaction in the presence of different organic solvents (figure 6*c*). Common organic solvents showed excellent conversion except alcohols (MeOH and EtOH) which exhibited the lowest conversion. An explanation for this phenomenon can be the large formation of H-bonding which diminishes the conversion of epoxidation [33]. Also, the reaction in aqueous conditions had a low efficiency (figure 6*c*) due to the formation of biphasic solution and mass-transfer problems. Dichloromethane and a mixture of dichloromethane and acetonitrile (1 : 1, 10 ml) demonstrated high conversion for epoxidation of styrene (figure 6*c*).

The results showed that the epoxide selectivity was completely independent of solvent or amount of dioxirane groups as indicated by green curves in figure 6. Epoxide selectivity remains almost constant during the reaction time along with effective parameters for epoxide conversion, which represents the high selectivity of PDOX for epoxidation of alkenes.

PDOX was used to carry out the epoxidation of a variety of olefins (table 3). A wide variety of substrates were consistent for PDOX epoxidation in the stepwise or *in situ* approach. As shown in table 3, the stepwise method exhibited a slightly better performance and shorter reaction times than *in situ* method. Also, the method demonstrated low side reaction as shown for most of the entries given in table 3. PDOX was completely chemoselective for epoxidation of terminal alkene than terminal α,β-unsaturated alkene demonstrated for 2-methylhexa-1,5-dien-3-one (table 3, entry 9). The resonance with carbonyl group results in the decrease of electron density on the α,β-unsaturated bond and so the epoxidation with PDOX selectively occurred on unconjugated double bond. Chemoselectivity behaviour of PDOX was also shown by epoxidation of one alkene group in the substrates with two alkene groups (table 3, entries 6, 10). Furthermore, as shown in optimization studies, high selectivity was achieved for all substrates (94–99%).

**Table 3.** Epoxidation of alkenes by PDOX^a^.

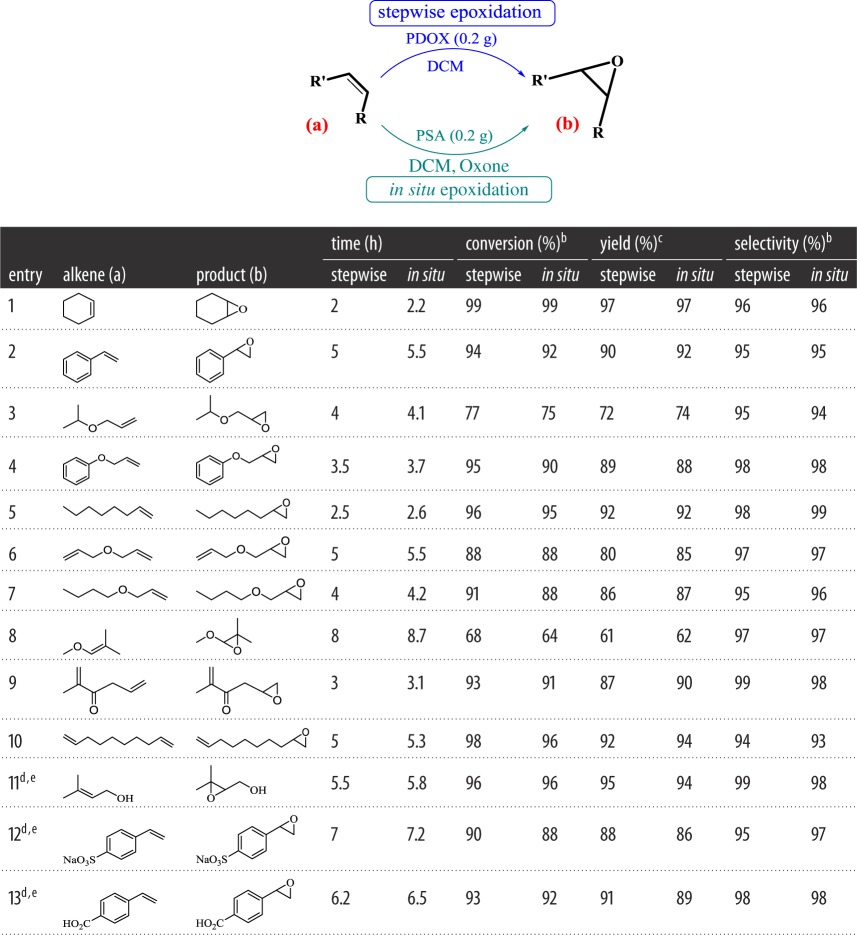

^a^Reaction conditions: stepwise epoxidation: 0.3 g PDOX (containing 2.25 mmol dioxirane group), DCM (10 ml) (water for 11b–13b), alkene (2 mmol), room temperature. *In situ* epoxidation: alkene (2.0 mmol), CH_2_Cl_2_ (25.0 ml), tetra-*n*-butylammonium hydrogen sulfate (10.0 mg) 0.3 g of PSA followed by a saturated solution of NaHCO_3_ was added to the reaction mixture. Oxone powder as oxygen source (1.9 g, 3 mmol) was added in portions.

^b^GC analysis.

^c^Isolated yield.

^d^Solvent: water (10 ml).

^e^When the reaction was completed, the catalyst was filtered off and then water (20 ml) was added and the mixture was extracted with ethyl acetate (5 × 20 ml). The organic layer was then washed with brine (2 × 15 ml), dried over sodium sulfate, filtered, and the ethyl acetate was removed by rotary evaporator to give the desired product.

**Scheme 2.**
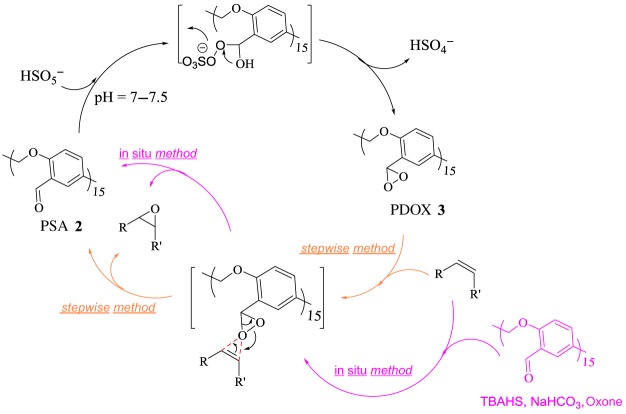
Plausible mechanism for *in situ* or stepwise epoxidation of olefins catalysed by PDOX **3**.

Steric hindrance extremely affects the epoxide conversion; for instance, the conversion for 1-methoxy-2,2-dimethyloxirane (table 3, entry 8) was 68%. Styrene shows more epoxidation efficiency and selectivity than cyclohexene (table 3, entries 1, 2). These results suggest that there is one more trend for epoxidation of *cis* alkene with PDOX due to spiro-transition state (as seen in epoxidation reactions with DMDO [32]), which prevails over its steric hindrance related to cyclohexene as shown for epoxidation of styrene and cyclohexene (table 3, entries 1, 2). Furthermore, the presence of oxygen in substrates with allyloxy moiety (table 3, entries 3, 6–8) decreases the electron density on the double bond and subsequently reaction conversion is reduced. The lack of epoxidation of α,β-unsaturated alkene with PDOX and above-mentioned evidence were in agreement with the presented reaction mechanism in scheme 2. As shown in scheme 2, selective dioxirane-mediated epoxidation of alkenes was performed by the formation of dioxirane groups on heterogeneous PSA (PDOX) through nucleophilic attack of HSO_5_^−^ to the aldehyde groups on PSA. Then oxygen was transferred from dioxirane groups on PDOX, through a spiro-transition state, to double bond followed by regeneration of PSA (scheme 2).

Also, the possibility of this method in aqueous media was investigated. For this goal, three water-soluble alkenes were epoxidized with PDOX in the presence of water as solvent and high yield and selectivity for epoxides were obtained (table 3, entries 11–13). The results indicated the capability and compatibility of PDOX for efficient epoxidation of water-soluble alkenes in water.

The ability to maintain PDOX, to prepare and use it as before, is an advantage in the stepwise method. On the other hand, the *in situ* method is a one-step process that does not require pre-PDOX preparation, but unlike the stepwise process, it requires pH adjustment to prepare *in situ* PDOX. Moreover, gas formation due to the addition of Oxone in the *in situ* method may cause problems concerning large-scale epoxidation of alkenes. Furthermore, the results of various epoxidations of alkenes (table 3) show that the stepwise method is better than *in situ* method in terms of the time and efficiency, while both methods provide the same selectivity.

Finally, we can count some advantages for this system over DMDO which are listed in table 4. PDOX can be easily prepared with simple and commercially accessible and safe materials and also isolated as a stable powder which can be kept in a refrigerator. PDOX can accomplish epoxidation of alkenes without the need for transition metal systems, toxic oxidants, highly toxic solvents, high temperature or complicated processes.

**Table 4.** Some advantages of PDOX over DMDO.

PDOX	DMDO
easy preparation	harsh and tedious procedures for preparation
possibility of isolation as stable powder	isolation must be as acetone solution at low temperatures and is largely unstable [27]
easy separation from the reaction mixture	it is homogeneous and leads to wasting acetone for every reaction
recoverability and reusability in consecutive runs without any significant reactivity loss	recovery needs distillation of acetone
easy characterization	difficult characterization (mostly need labelled acetone) [32]

**Figure 7.**
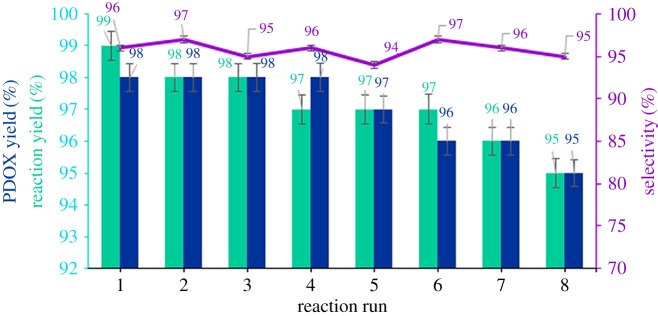
Recycling studies on epoxidation of styrene catalysed by PDOX.

Another positive point of this work was the recovery ability of PDOX which not only highlighted this method but also demonstrated the stability of PDOX during the reaction. The recovery ability of PSA (as a PDOX precursor) was studied in defined model reaction. Recovered PDOX was treated with hot water to complete decomposition of remaining dioxirane groups. Recovered PSA was characterized and its structure was proved by FTIR, CHN and NMR analyses and compared with original spectra (after each run) then transformed to PDOX and used in the next run. The experiments showed that PDOX can be recovered from the reaction mixture for eight consecutive runs, wherein yield of PDOX, yield of epoxidation or epoxide selectivity did not show any significant drop (figure 7).

## 5. Conclusion

We have developed a new heterogeneous PDOX as an efficient oxidant for epoxidation of olefins, which overcomes large drawbacks concerning DMDO. Simple preparation method, ease of isolation as stable powder, recyclability, efficient epoxidation of alkenes, simple work-up procedure, *in situ* or stepwise preparation were some of the advantages of PDOX that should be considered. Furthermore, being environment-friendly and economical, this method is a suitable alternative for DMDO. PDOX demonstrated good performance for epoxidation of water-soluble alkenes in aqueous media. We are also looking forward to introduce more applications for PDOX.

## Supplementary Material

Supplementary Information

## Data accessibility

All data that are relevant to the study are reported within the article. Also, the datasets supporting this article have been uploaded as part of the electronic supplementary material.
