# Supplementary material for: Selective and metal-free epoxidation of terminal alkenes by heterogeneous polydioxirane in mild conditions
Source: R Soc Open Sci. 2018 May 2;5(5):171541. doi: 10.1098/rsos.171541 (PMC5990723; doi:10.1098/rsos.171541)

**Supplementary information**

Selective, metal free epoxidation of terminal alkenes by heterogeneous polydioxirane (PDOX) in mild conditions

M. Kazemnejadi,^a^ A. Shakeri,*^b^ M. Nikookar,^a^ R. Shademani and M. Mohammadi^a^

*^a^ Department of Chemistry, College of Sciences, Golestan University, Gorgan, Iran.*

**^b^ Faculty of Chemistry, University College of Science, University of Tehran, Tehran, Iran. Email: Alireza.shakeri@khayam.ut.ac.ir; Tel: +989111754806.*

*Department of Marine Technology, Amirkabir University of Technology, Tehran, Iran*

**Table of Contents**

**1. Characterization data for oxirane derivatives S2**

**1.1. General synthesis for Stepwise PDOX epoxidation of alkenes S2**

**1.2. General synthesis for *in situ* PDOX epoxidation of alkenes S2**

**1.3. Physical and spectral data for oxirane derivatives S2**

**1.3.1. 7-oxabicyclo[4.1.0]heptane (1b) S2**

**1.3.2. Epoxy styrene (2b) S3**

**1.3.3. 2-(isopropoxymethyl)oxirane (3b) S3**

**1.3.4. 2-(phenoxymethyl)oxirane (4b) S3**

**1.3.5. 2-hexyloxirane (5b) S3**

**1.3.6. 2-((allyloxy)methyl)oxirane (6b) S4**

**1.3.7. 2-(butoxymethyl)oxirane (7b) S4**

**1.3.8. 3-methoxy-2,2-dimethyloxirane (8b) S4**

**1.3.9. 3-methyl-1-(oxiran-2-yl)but-3-en-2-one (9b) S4**

**1.3.10. 2-(oct-7-en-1-yl)oxirane (10b) S5**

**1.3.11. (3,3-dimethyloxiran-2-yl)methanol (11b) S5**

**1.3.12. Sodium 4-(oxiran-2-yl)benzenesulfonate (12b) S5**

**1.3.13. 4-(oxiran-2-yl)benzoic acid (13b) S5**

**1.4. ^13^C NMR and ^1^H NMR spectra of oxirane derivatives S6**

**1. Characterization data for oxirane derivatives:**

**1.1. General synthesis for Stepwise PDOX epoxidation of alkene:**

**1b-10b:** In a typical run for stepwise epoxidation of alkenes by PDOX **3**, to a 25 mL round bottom flask, 0.3 g of PDOX (containing 2.25 mmol dioxirane group) was added to an anhydrous CH_2_Cl_2_ (10 mL) solution of alkene (2.0 mmol). The mixture was stirred at room temperature (heterogeneous medium). After completion of the reaction as indicated by TLC (2 - 8 h), the catalyst was filter off and the solvent was evaporated to give epoxide product. Flash chromatography was used in some cases for further purification. The recovered reagent (that is now PSA) was washed with distilled water (3×5 mL) and acetone (3×5 mL) and stored in the refrigerator for next reaction.

**1.2. General synthesis for *in situ* PDOX epoxidation of alkene:**

**1b-10b:** Epoxidation of alkenes was also accomplished by *in situ* preparation of PDOX in the mixture as following described procedure: Alkene (2.0 mmol) was dissolved in 25.0 mL of CH_2_Cl_2_. Then 10 mg of tetra-*n*-butylammonium hydrogen sulfate was dissolved in water (20 mL) and added to the reaction mixture. pH value of the reaction mixture was monitored and adjusted to 7.5 by addition of phosphate buffer (10 mL). 0.3 g of PSA followed by a saturated solution of NaHCO_3_ was added to the reaction mixture. Oxone powder as oxygen source (1.9 g, 3 mmol) was added in portions. The reaction mixture was stirred vigorously while maintaining the pH of the solution at 7.0-7.5 (Heterogeneous medium). Upon completion of the reaction monitored by TLC, PSA was filtered and the remaining solution poured into a separate funnel and diluted with one volume of ethyl acetate. The layers were separated and the organic layer was washed with H_2_O, dried over K_2_CO_3_, then evaporated at reduced pressure (Aqueous layer was discarded). The crude product was purified by flash chromatography on silica pretreated with triethylamine (TEA) using dry loading.

**11b-13b:** For these compounds water was used as solvent (other reaction conditions were same as before). When the reaction was completed, PSA was filtered and then water (20 mL) was added and the mixture was extracted with ethyl acetate (5×20 mL). The organic layer was then washed with brine (2×15 mL), dried over sodium sulfate, filtered, and the ethyl acetate was removed by rotary evaporator to give the desired product.

**1.3. Physical and spectral data for oxirane derivatives**

The reported conversion and yield are according to stepwise results.

**1.3.1. 7-oxabicyclo[4.1.0]heptane (1b)**

Colorless liquid; Conversion (GC): 99 %; Yield = 97 %; **^1^H NMR** (300 MHz, CDCl_3_) δ (ppm): 0.72-0.80 (m, 2H), 0.87-0.96 (m, 2H), 1.30-1.46 (m, 2H), 2.56 (m, 2H); **^13^C NMR** (75 MHz, CDCl_3_): δ (ppm) = 20.4, 25.2, 53.2; **CHN**: Found %: C 73.45; H 10.87. C_6_H_10_O. Calculated, %: C 73.43; H 10.27.

**1.3.2. Epoxy styrene (2b)**

Colorless liquid; Conversion (GC): 94 %; Yield = 90 %; **^1^H NMR** (300 MHz, CDCl_3_) δ (ppm): 2.73-3.03 (m, 2H), 4.21-4.28 (m, 1H), 7.43 (s, 5H); **^13^C NMR** (75 MHz, CDCl_3_): δ (ppm) = 50.4, 51.3, 124.6, 128.7, 130.0, 135.8; **CHN**: Found %: C 79.91; H 6.32. C_8_H_8_O. Calculated, %: C 79.97; H 6.71.

**1.3.3. 2-(isopropoxymethyl)oxirane (3b)**

Colorless liquid; Conversion (GC): 77 %, Yield = 72 %; **^1^H NMR** (300 MHz, CDCl_3_): δ (ppm) = 0.57 (dd, *J_1_*= 2.75, *J_2_*=6.00, 6H), 1.97-2.00 (m, 1H), 2.14-2.18 (m, 1H), 2.75-2.81 (m, 1H), 3.06-3.11 (m, 2H); **^13^C NMR** (75 MHz, CDCl_3_): δ (ppm) = 22.9, 44.0, 52.3, 68.9, 75.8; **CHN**: Found %: C 61.98; H 10.27. C_6_H_12_O_2_. Calculated, %: C 62.04; H 10.41.

**1.3.4. 2-(phenoxymethyl)oxirane (4b)**

Colorless liquid; Conversion (GC): 95 %, Yield = 89 %; **^1^H NMR** (300 MHz, CDCl_3_): δ (ppm) = 2.61-2.64 (m, 1H), 2.77 (t, *J*=4.25, 1H), 3.24-3.27 (m, 1H), 3.75-3.82 (m, 1H), 4.13 (dd, *J_1_*=3.00 ,*J_2_*=11.24 ,1H), 6.90-6.96 (m, 3H), 7.28 (t, *J*=8.50); **^13^C NMR** (75 MHz, CDCl_3_): δ (ppm) = 44.6, 50.2, 69.2, 114.5, 121.3, 130.3, 158.5; **CHN**: Found %: C 71.47; H 6.87. C_9_H_10_O_2_. Calculated, %: C 71.98; H 6.71.

**1.3.5 2-hexyloxirane (5b)**

Colorless liquid; Conversion (GC): 96 %, Yield = 92 %; **^1^H NMR** (300 MHz, CDCl_3_): δ (ppm) = 2.44-2.47 (m, 3H), 2.63-2.66 (m, 3H), 2.97-3.03 (m, 3H), 3.23, 3.30 (m, 3H), 3.34-3.50 (m, 5H); **^13^C NMR** (75 MHz, CDCl_3_): δ (ppm) = 14.0, 22.1, 26.5, 28.8, 31.9, 32.5, 48.6, 52.1; **CHN**: Found %: C 74.51; H 12.77. C_8_H_16_O. Calculated, %: C 74.94; H 12.58.

**1.3.6. 2-((allyloxy)methyl)oxirane (6b)**

Colorless liquid; Conversion (GC): 96 %, Yield = 92 %; **^1^H NMR** (300 MHz, CDCl_3_): δ (ppm) = 2.31-2.35 (m, 1H), 2.49-2.53 (m, 1H), 2.87-2.88 (m, 1H), 3.08-3.15 (m, 1H), 3.46-3.52 (m, 1H), 3.77-3.82 (m, 2H), 4.90 (d, *J*= 12.50, 1H), 5.00 (d, *J*= 12.50, 1H), 5.62-5.73 (m, 1H); **^13^C NMR** (75 MHz, CDCl_3_): δ (ppm) = 13.3, 18.3, 31.7, 45.4, 49.7, 71.8; **CHN**: Found %: C 63.84; H 8.83. C_6_H_10_O­_2_. Calculated, %: C 63.14; H 8.83.

**1.3.7. 2-(butoxymethyl)oxirane (7b)**

Colorless liquid; Conversion (GC): 91 %, Yield = 86 %; **^1^H NMR** (300 MHz, CDCl_3_): δ (ppm) = 0.34 (t, *J*= 7.50, 3H), 0.76-1.10 (m, 2H), 1.95-1.98 (m, 1H), 2.12-2.16 (m, 1H), 2.49-2.52 (m, 1H), 2.71-2.78 (m, 1H), 2.88-2.93 (m, 2H), 3.07-3.10 (m, 1H); **^13^C NMR** (75 MHz, CDCl_3_): δ (ppm) = 13.3, 18.3, 31.7, 45.4, 49.7, 71.8; **CHN**: Found %: C 64.24; H 10.28. C_6_H_10_O_2_. Calculated, %: C 64.58; H 10.84.

**1.3.8. 3-methoxy-2,2-dimethyloxirane (8b)**

Colorless liquid; Conversion (GC): 68 %, Yield = 61 %; **^1^H NMR** (300 MHz, CDCl_3_) δ (ppm): 0.89 (s, 3H), 0.98 (s, 3H), 3.14 (s, 3H), 3.94 (s, 1H); **^13^C NMR** (75 MHz, CDCl_3_) δ (ppm): 20.9, 57.3, 59.2, 96.6; **CHN**: Found %: C 58.30; H 9.78. C_5_H_10_O_2_. Calculated, %: C 58.80; H 9.87.

**1.3.9. 3-methyl-1-(oxiran-2-yl)but-3-en-2-one (9b)**

Colorless liquid; Conversion (GC): 93 %, Yield = 87 %; **^1^H NMR** (300 MHz, CDCl_3_) δ (ppm): 1.66 (s, 3H), 2.34-2.37 (m, 1H), 2.53 (t, *J*=4.25), 2.93-2.96 (m, 1H), 3.66 (dd, *J_1_*=3.00 , *J_2_=*12.25*,* 1H), 4.18 (dd, *J_1_*=2.5, *J_2_=*12.5*,* 1H), 5.33 (s, 1H), 5.85 (s, 1H); **^13^C NMR** (75 MHz, CDCl_3_) δ (ppm): 116.31, 119.31, 123.35, 128.84, 129.33, 156.45, 164.28; **CHN**: Found %: C 66.13; H 7.24. C_7_H_10_O_2_. Calculated, %: C 66.65; H 7.92.

**1.3.10. 2-(oct-7-en-1-yl)oxirane (10b)**

Colorless liquid; Conversion (GC): 98 % Yield = 96 %; **^1^H NMR** (300 MHz, CDCl_3_) δ (ppm): 1.30-1.48 (m, 10H), 1.99 (m, 2H), 2.44 (m, 1H), 2.72 (m, 1H), 2.87 (m, 1H), 4.86-4.99 (m, 1H), 5.22-5.29 (m, 1H), 5.68-5.80 (m, 1H); **^13^C NMR** (75 MHz, CDCl_3_) δ (ppm): 26.8, 28.1, 29.3, 30.0, 31.7, 33.6, 47.0, 52.3, 114.1, 138.8; **CHN**: Found %: C 77.88; H 11.98. C_10_H_18_O. Calculated, %: C 77.87; H 11.76.

**1.3.11.** **(3,3-dimethyloxiran-2-yl)methanol (11b)**

Colorless liquid; Conversion (GC): 96 %; Yield = 96 %; **^1^H NMR** (300 MHz, DMSO-*d_6_*) δ (ppm): 1.24 (S, 6H), 2.13-2.19 (t, *J*= 7.25, 1H), 2.63 (s, 1H), 3.40-3.55 (m, 2H); **^13^C NMR** (75 MHz, DMSO-*d_6_*) δ (ppm): 22.4, 61.5, 62.6, 69.7; **CHN**: Found %: C 58.21; H 9.48. C_10_H_18_O. Calculated, %: C 58.80; H 9.87.

**1.3.12.** **Sodium 4-(oxiran-2-yl)benzenesulfonate (12b)**

Colorless liquid; Conversion (GC): 90 %, Yield = 88 %; **^1^H NMR** (300 MHz, DMSO-*d_6_*) δ (ppm): 2.74-3.22 (m, 2H), 4.05-4.08 (m, 1H), 7.52 (d, *J*= 7.85, 2H), 7.79 (d, *J*= 7.85, 2H); **^13^C NMR** (75 MHz, DMSO-*d_6_*) δ (ppm): 49.8, 56.6, 125.5, 126.6, 140.9, 148.9; **CHN**: Found %: C 43.57; H 11.98. C_10_H_18_O. Calculated, %: C 43.25; H 12.08.

**1.3.13.** **4-(oxiran-2-yl)benzoic acid (13b)**

Colorless liquid; Conversion (GC): 93 %; Yield = 92 %; **^1^H NMR** (300 MHz, DMSO-*d_6_*) δ (ppm): 2.61-3.18 (m, 2H), 3.45-3.52 (m, 1H), 7.24 (d, *J*= 7.77, 2H), 8.04 (d, *J*= 7.77, 2H); **^13^C NMR** (75 MHz, DMSO-*d_6_*) δ (ppm): 50.6, 54.2, 125.1, 129.7, 130.1, 143.4, 170.2; **CHN**: Found %: C 77.88; H 11.98. C_10_H_18_O. Calculated, %: C 77.87; H 11.76.

**1.3. ^13^C NMR and ^1^H NMR spectra of oxirane derivatives**


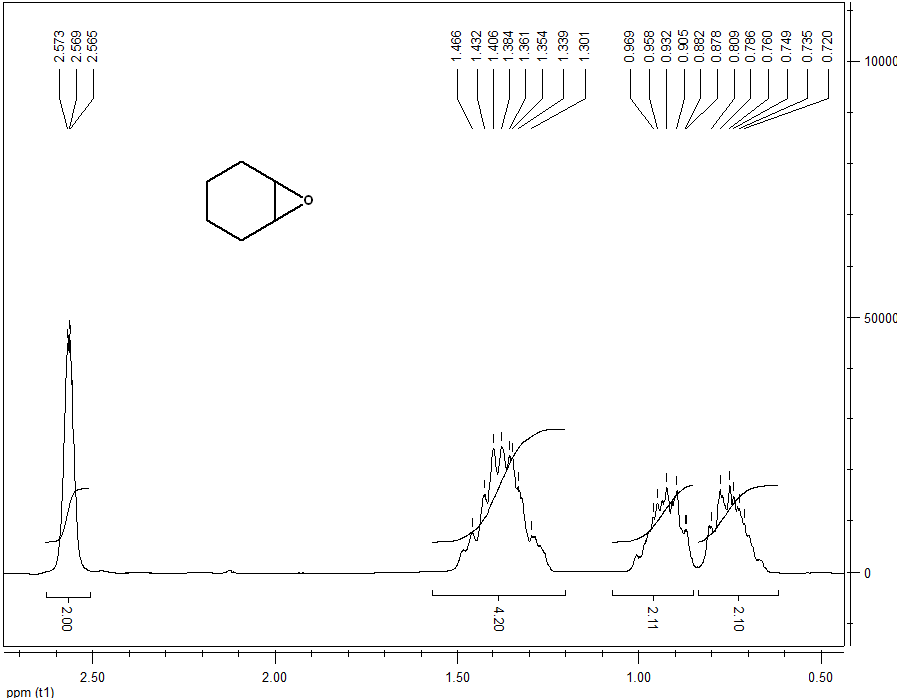


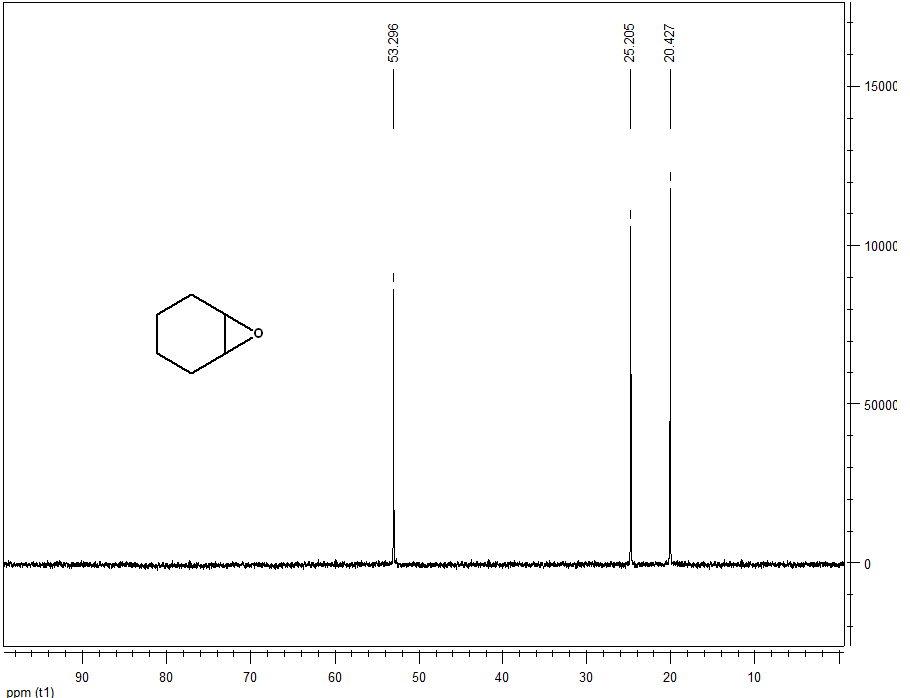


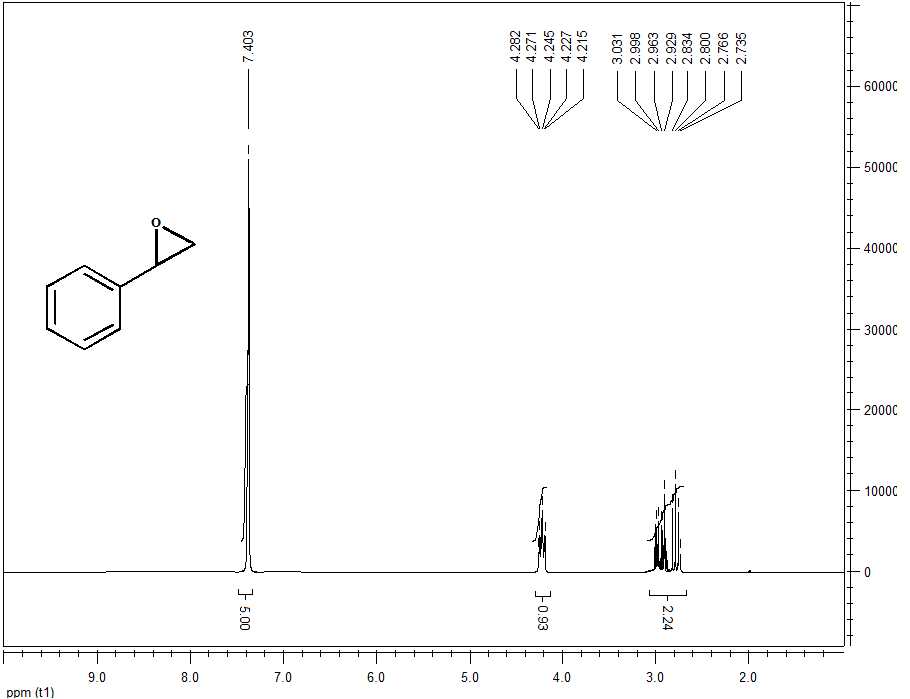


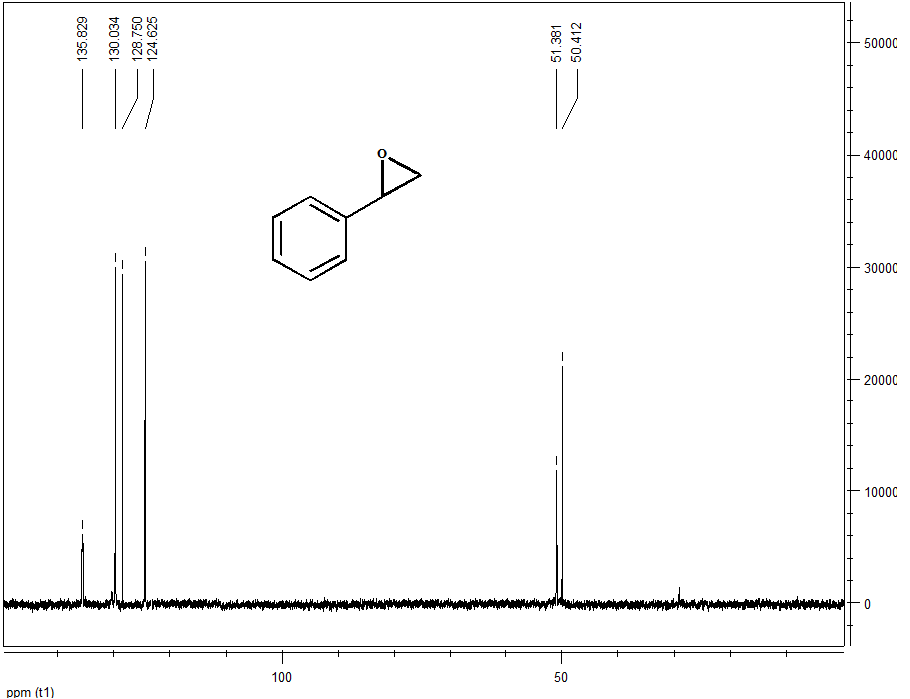


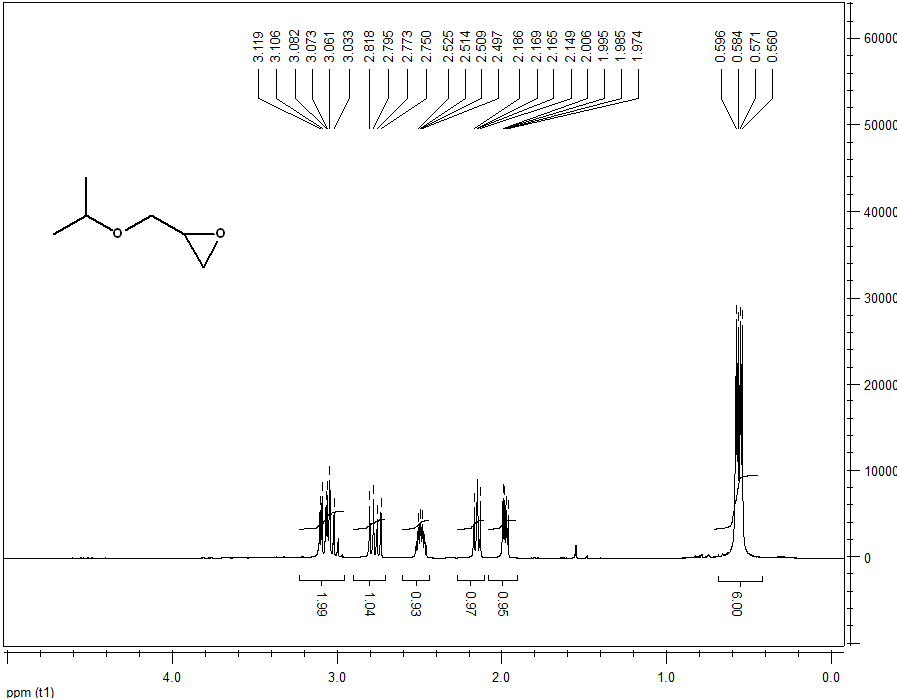


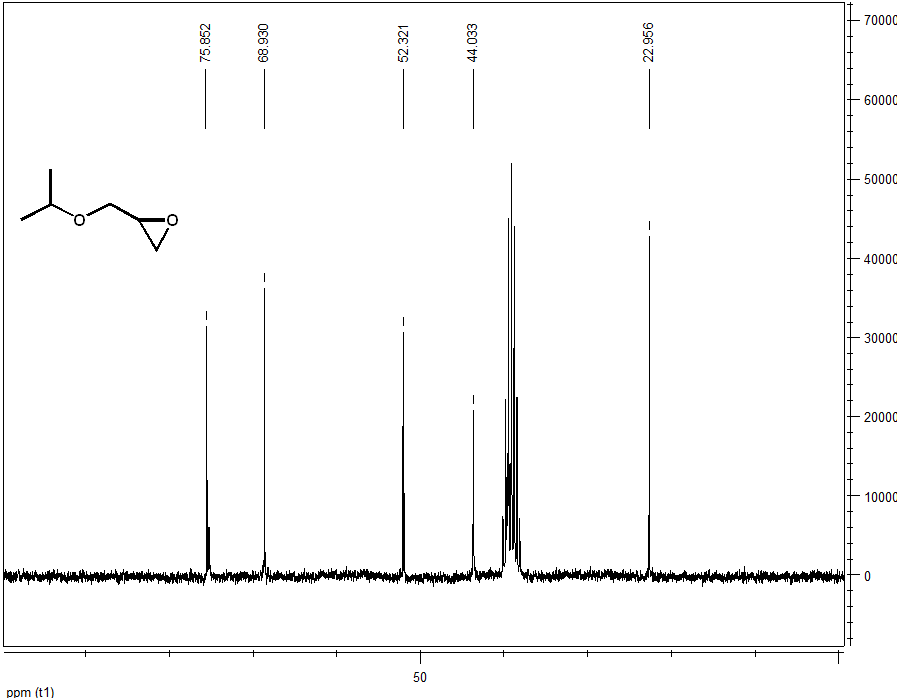


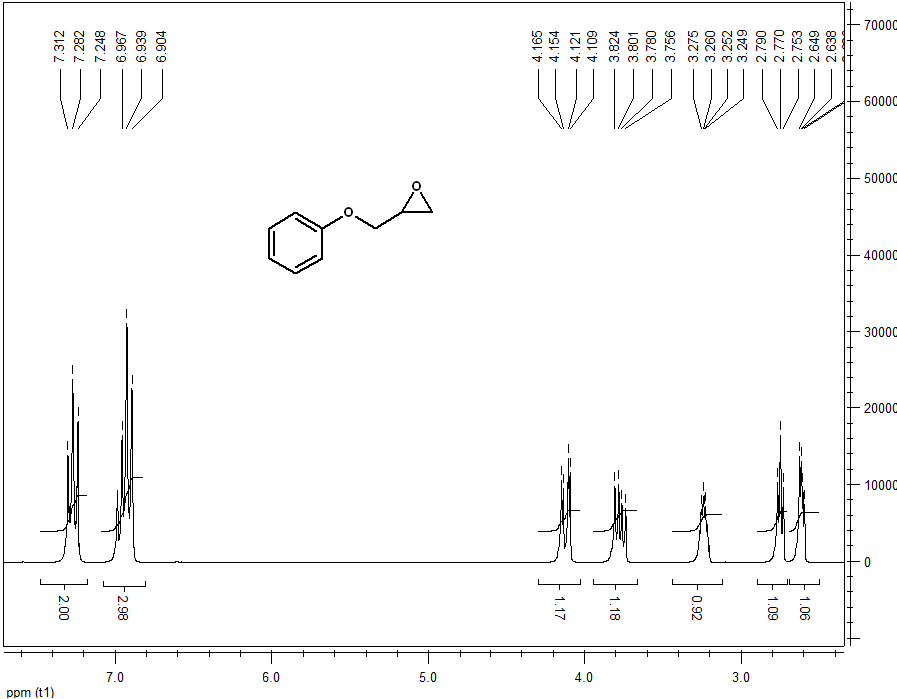


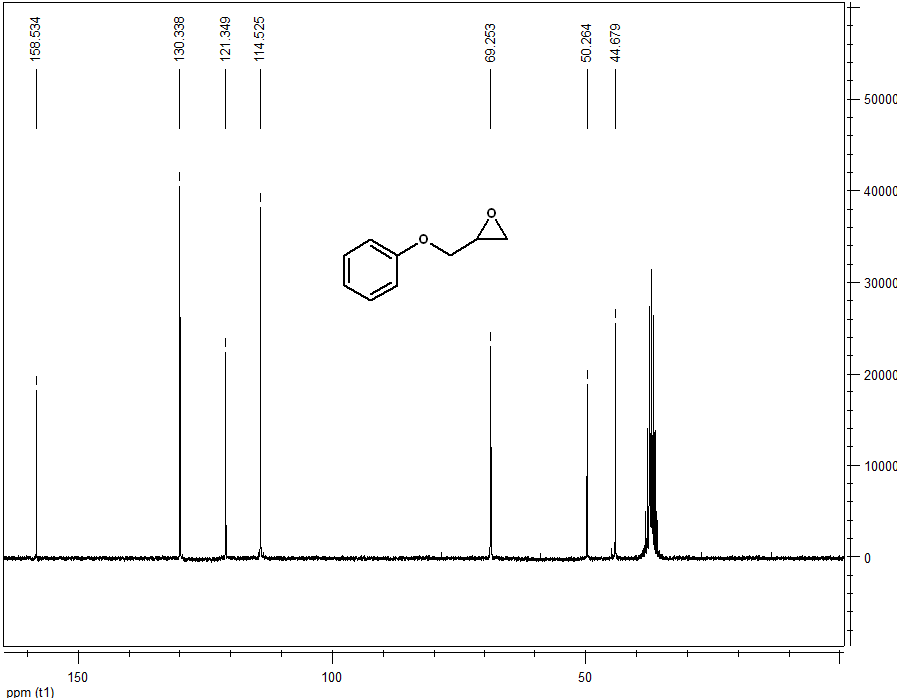


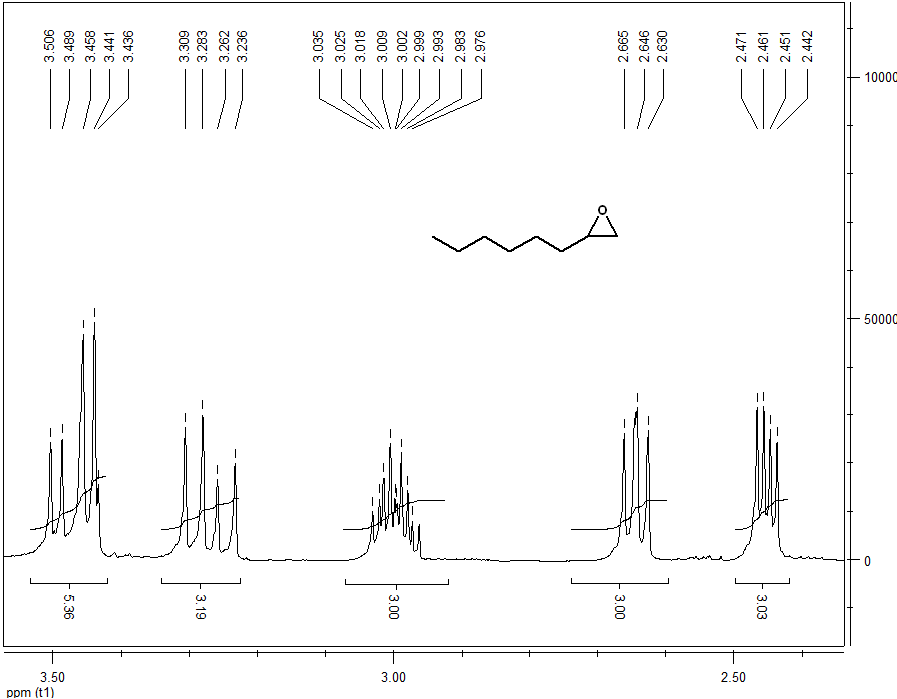


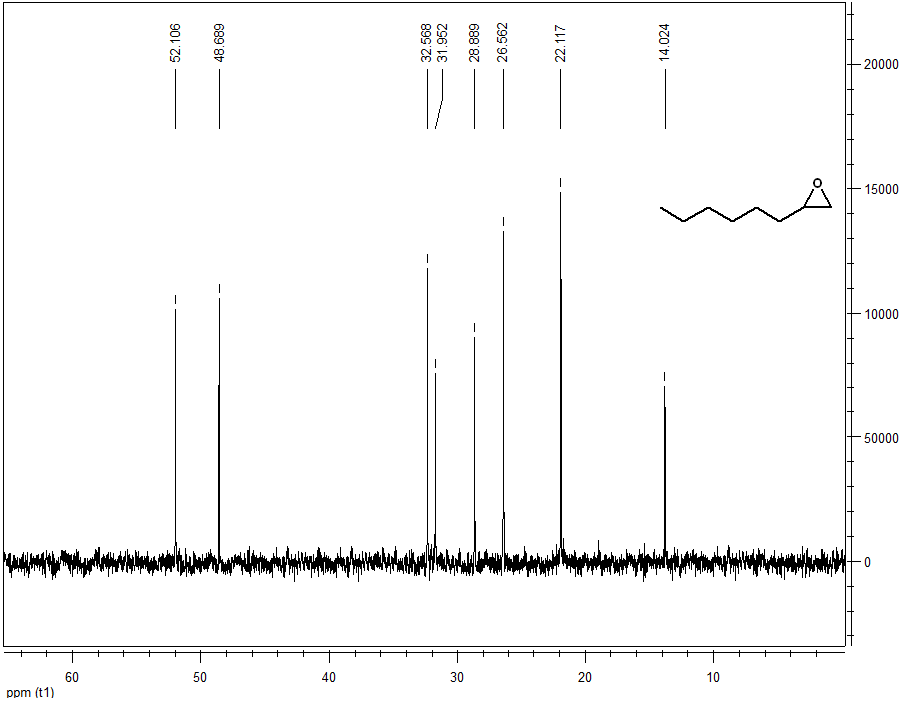


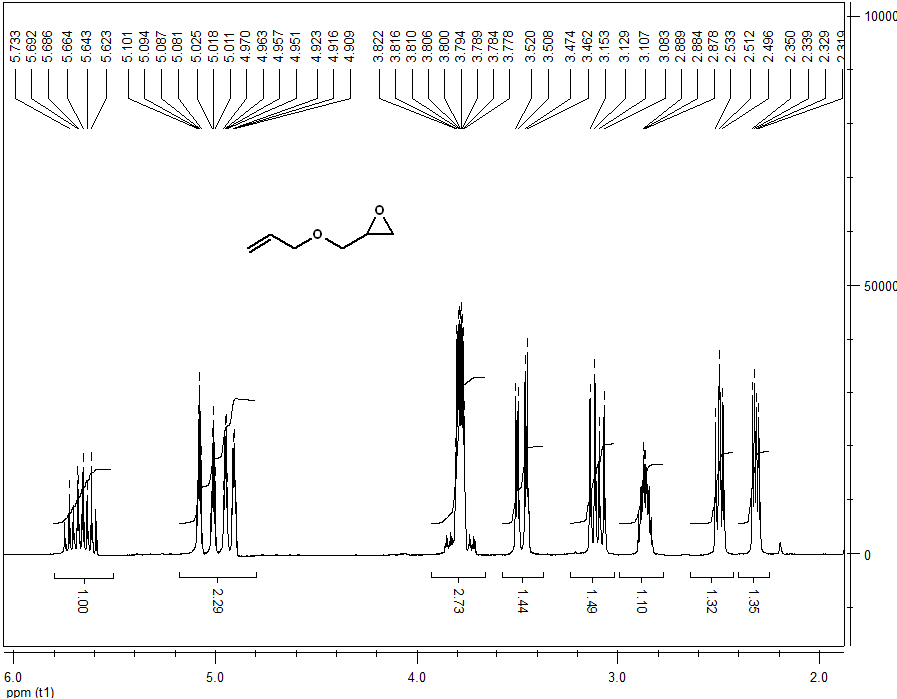


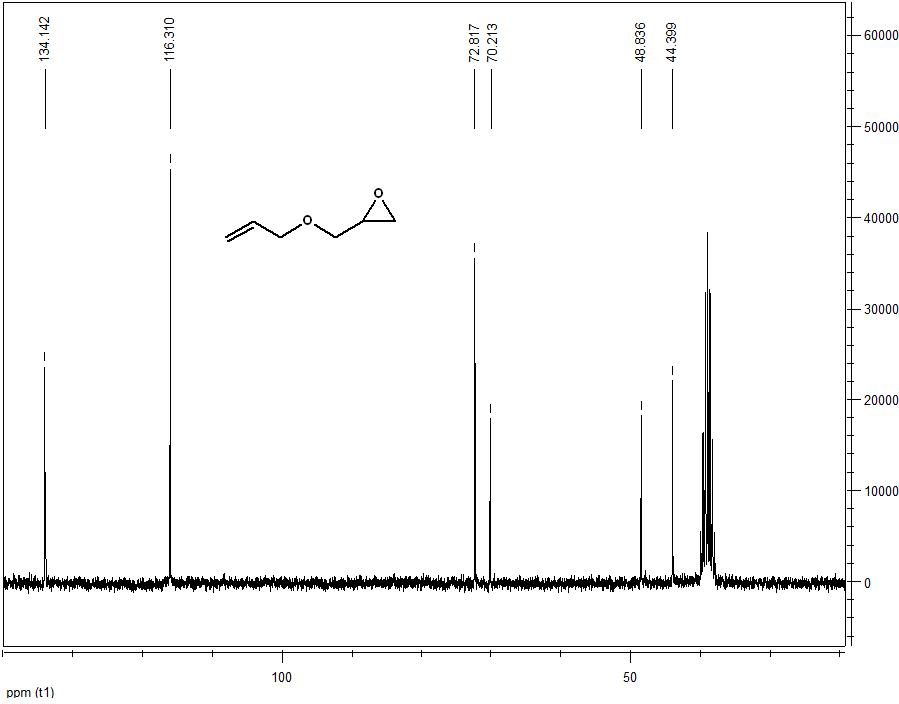


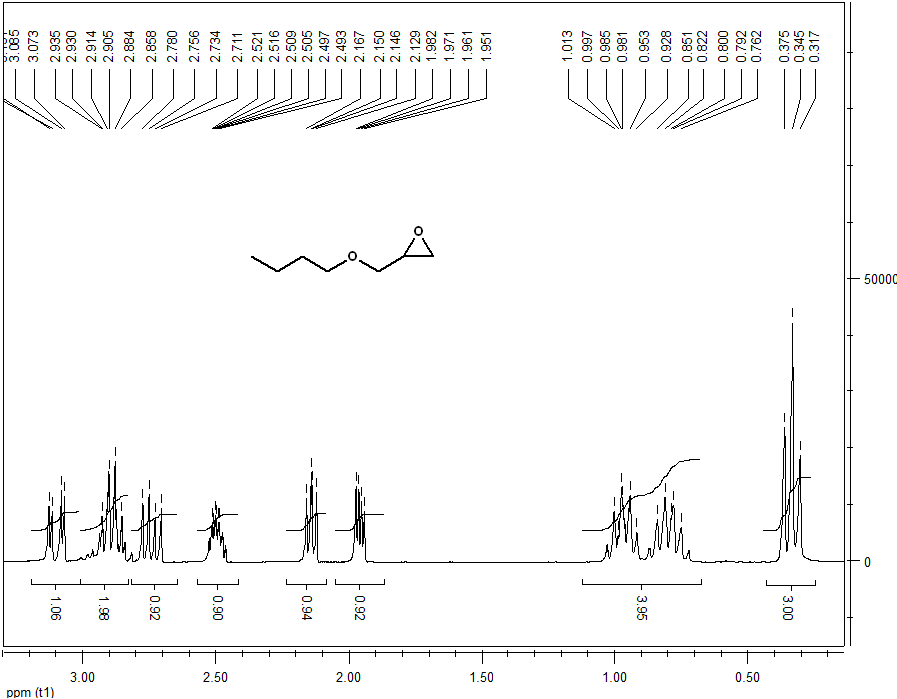


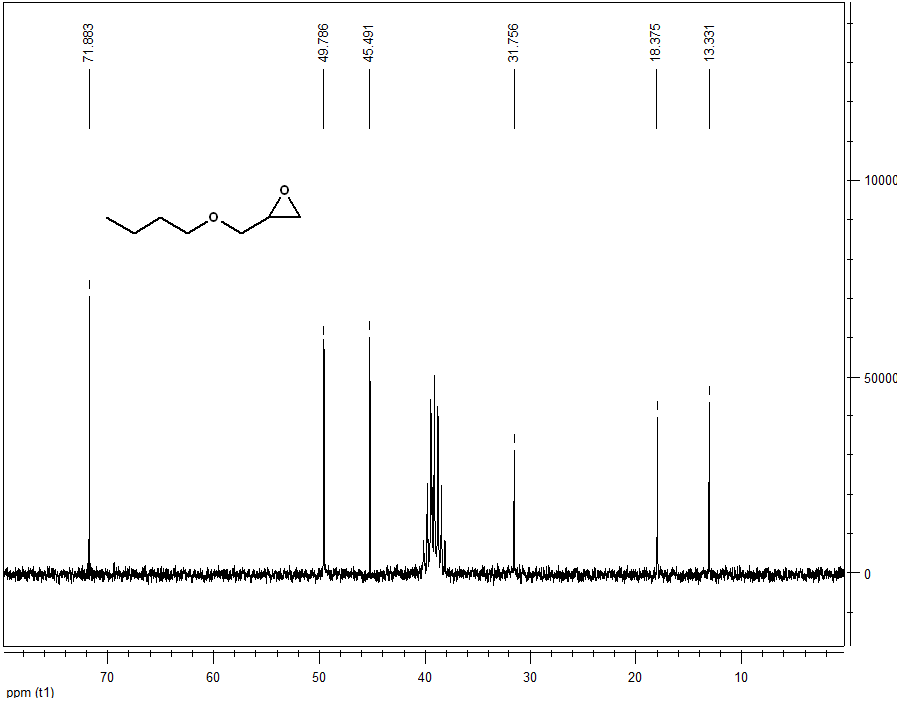


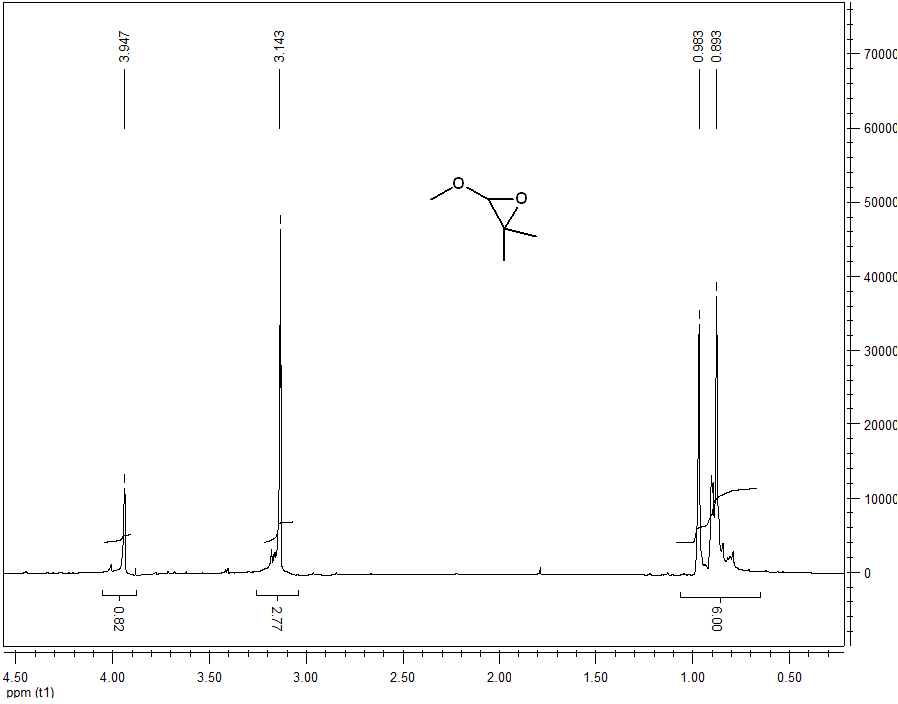


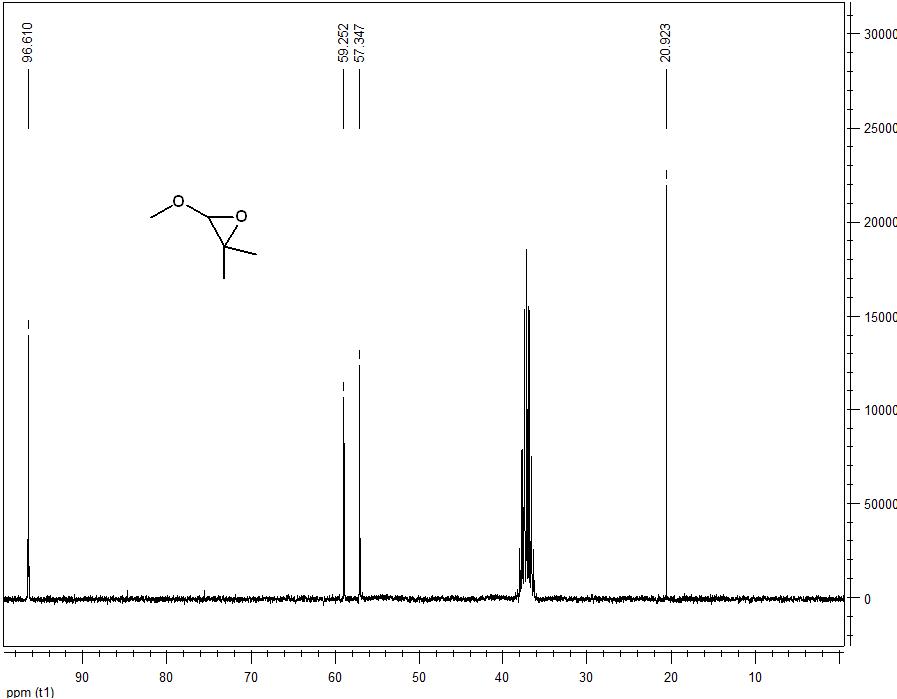


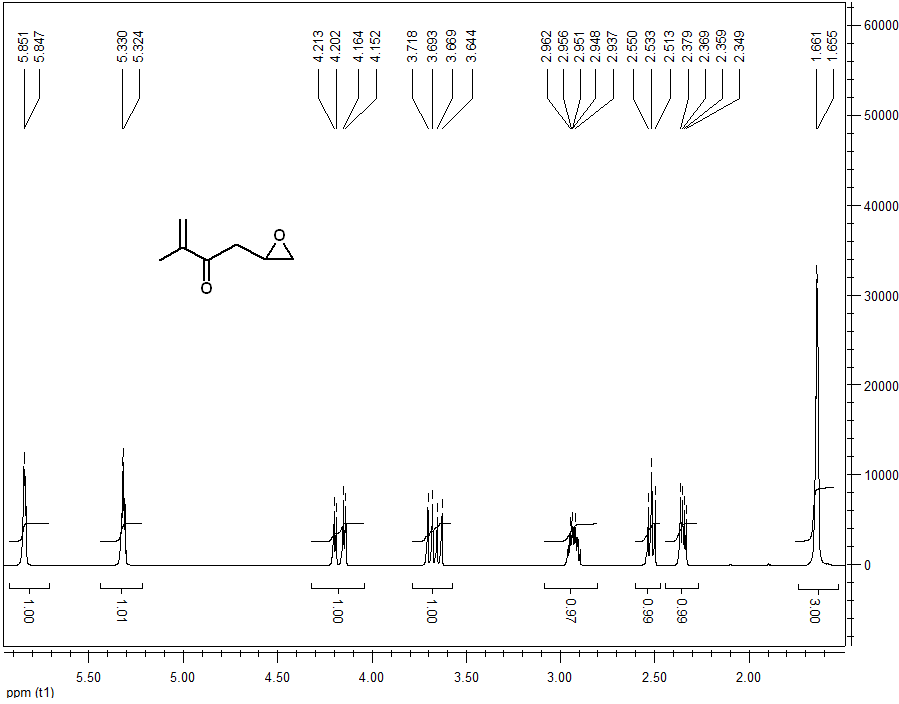


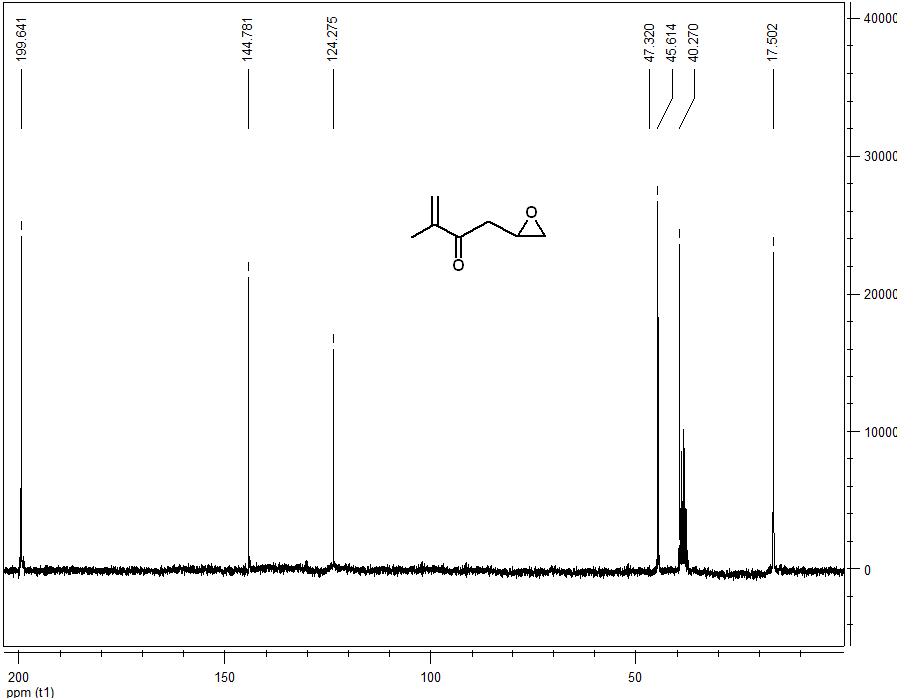


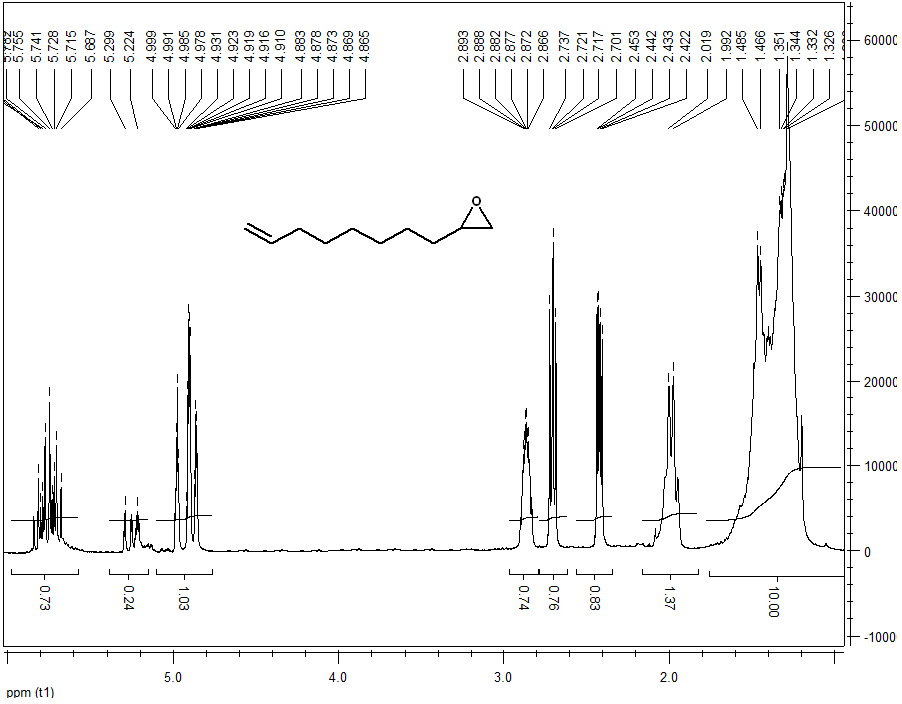


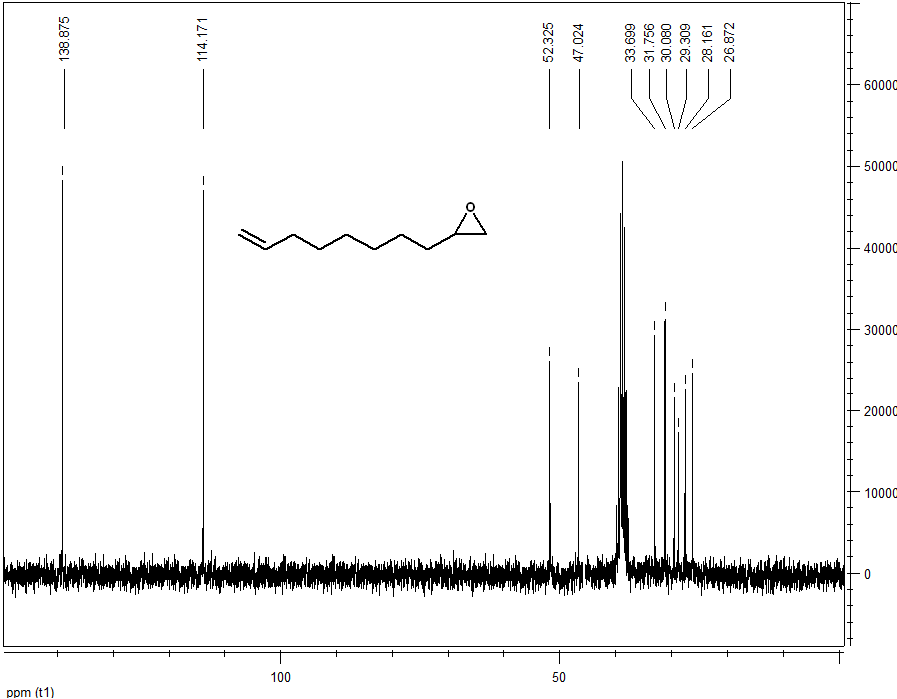


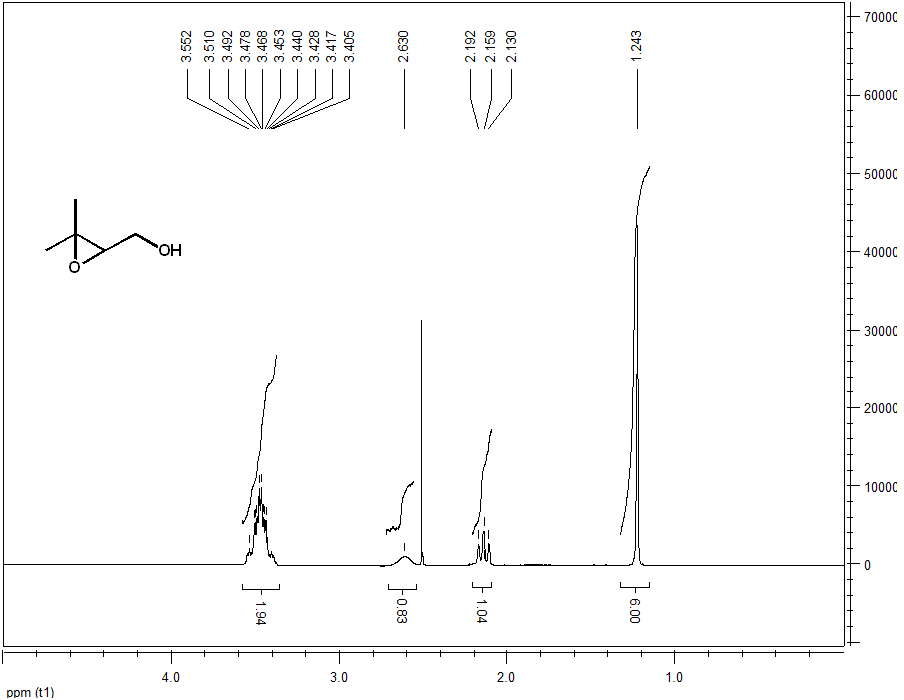


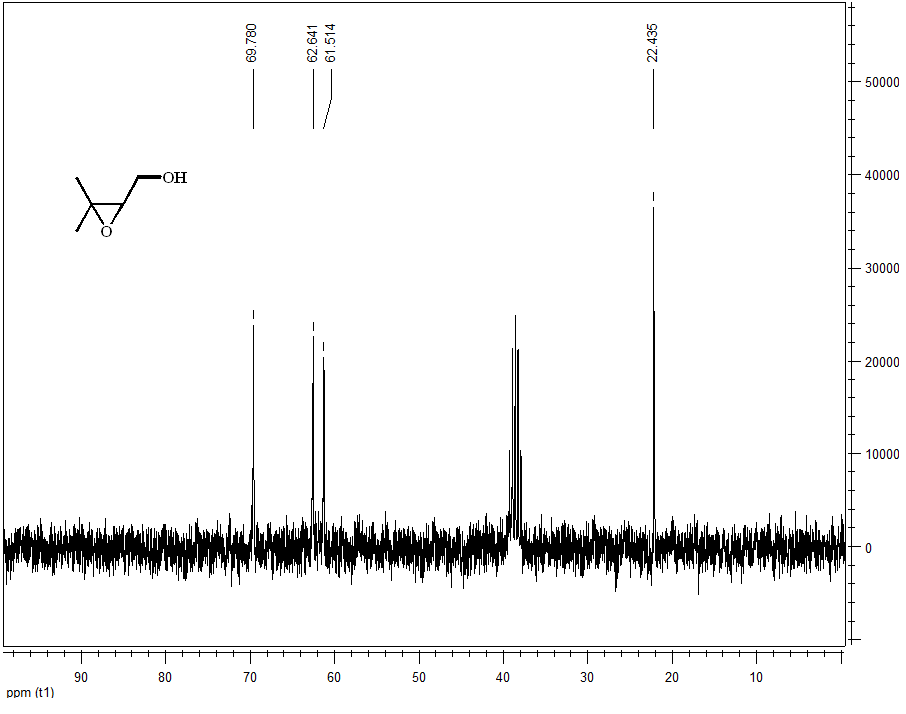


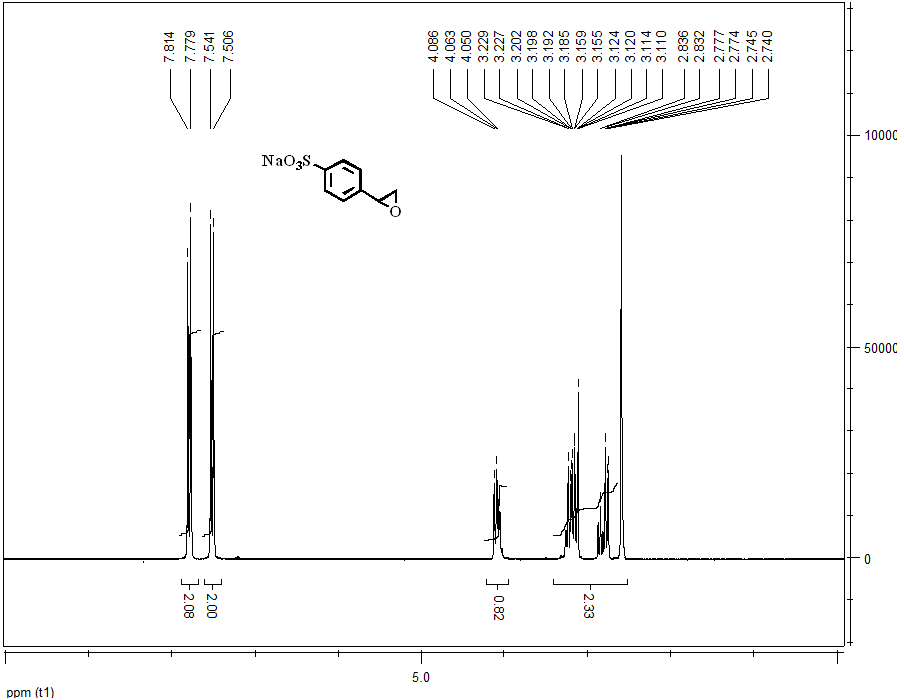


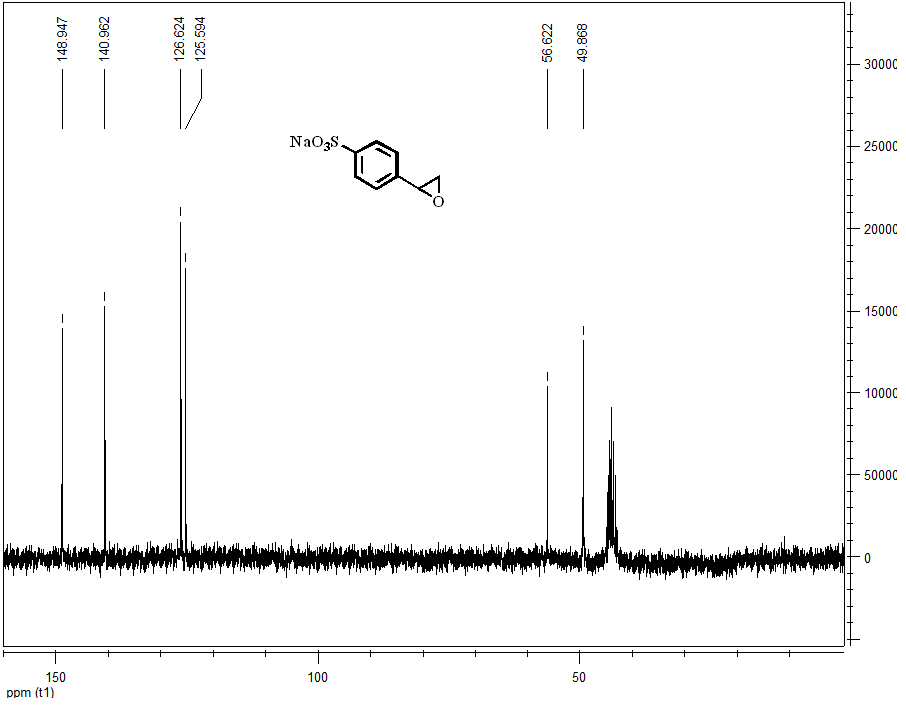


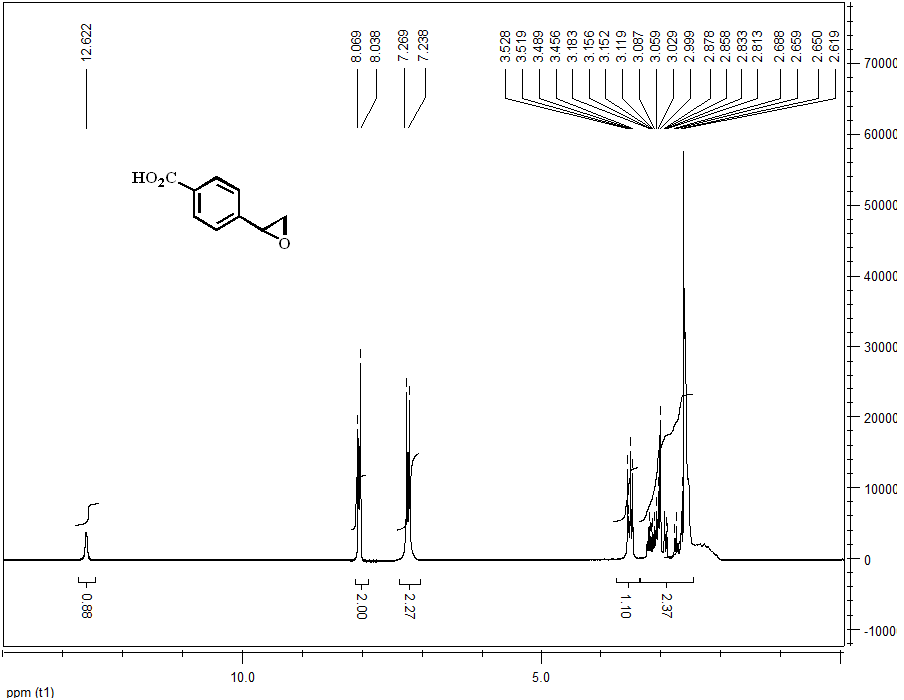


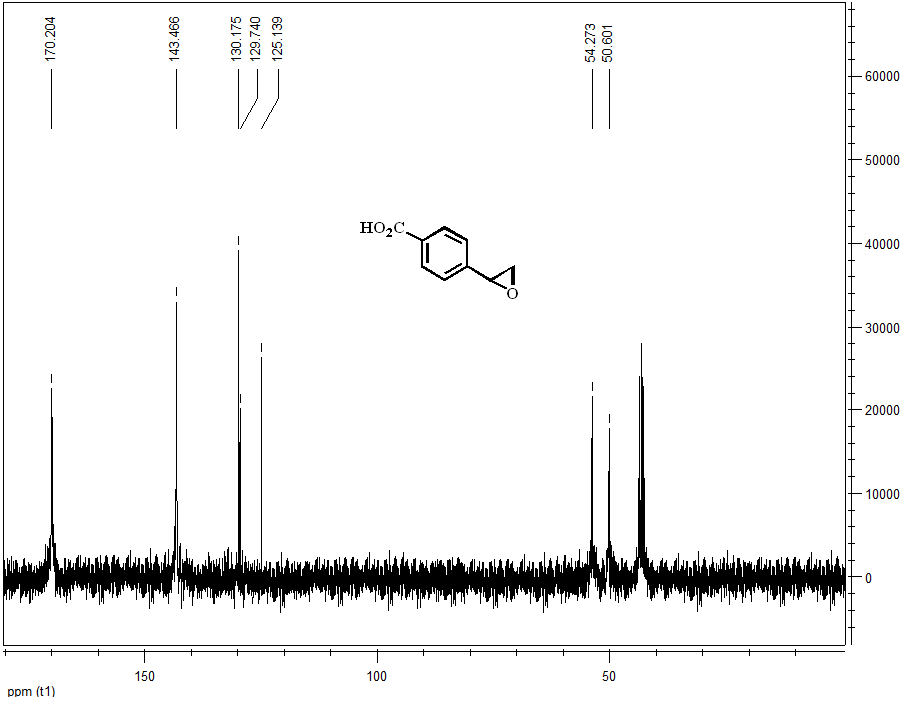

Supplement: Supplementary Information [file rsos171541supp1.doc]
